# Accumulation of Flavonols over Hydroxycinnamic Acids Favors Oxidative Damage Protection under Abiotic Stress

**DOI:** 10.3389/fpls.2016.00838

**Published:** 2016-06-15

**Authors:** Vicente Martinez, Teresa C. Mestre, Francisco Rubio, Amadeo Girones-Vilaplana, Diego A. Moreno, Ron Mittler, Rosa M. Rivero

**Affiliations:** ^1^Department of Plant Nutrition, Centro de Edafología y Biología Aplicada del Segura, Consejo Superior de Investigaciones CientíficasMurcia, Spain; ^2^Department of Food Science and Technology, Centro de Edafología y Biología Aplicada del Segura, Consejo Superior de Investigaciones CientíficasMurcia, Spain; ^3^Department of Biological Sciences, College of Arts and Sciences, University of North TexasDenton, TX, USA

**Keywords:** abiotic stress combination, salinity, heat, oxidative metabolism, metabolomics, phenylpropanoid metabolism, tomato

## Abstract

Efficient detoxification of reactive oxygen species (ROS) is thought to play a key role in enhancing the tolerance of plants to abiotic stresses. Although multiple pathways, enzymes, and antioxidants are present in plants, their exact roles during different stress responses remain unclear. Here, we report on the characterization of the different antioxidant mechanisms of tomato plants subjected to heat stress, salinity stress, or a combination of both stresses. All the treatments applied induced an increase of oxidative stress, with the salinity treatment being the most aggressive, resulting in plants with the lowest biomass, and the highest levels of H_2_O_2_ accumulation, lipid peroxidation, and protein oxidation. However, the results obtained from the transcript expression study and enzymatic activities related to the ascorbate-glutathione pathway did not fully explain the differences in the oxidative damage observed between salinity and the combination of salinity and heat. An exhaustive metabolomics study revealed the differential accumulation of phenolic compounds depending on the type of abiotic stress applied. An analysis at gene and enzyme levels of the phenylpropanoid metabolism concluded that under conditions where flavonols accumulated to a greater degree as compared to hydroxycinnamic acids, the oxidative damage was lower, highlighting the importance of flavonols as powerful antioxidants, and their role in abiotic stress tolerance.

## Introduction

Environmental conditions governing most of the agricultural lands worldwide are often inadequate for crop development and production. Abiotic stresses such as salinity, high temperature and water scarcity have been reported to cause large economic losses worldwide every year. Moreover, climatic predictions made by the IPCC (International Panel of Climate Change) for 2080 indicate a substantial deterioration of current conditions (IPCC 2014, http://www.ipcc.ch/). This, together with the expected increase in the world's population, highlight the urgent need for generating crops with increased tolerance to abiotic stress conditions. Traditionally, the effects of abiotic stress on plants have been studied through the application of a single stress, such as salinity, drought or heat, under controlled laboratory conditions (Hirayama and Shinozaki, 2010; Chew and Halliday, 2011). However, many agricultural lands affected by salinity are also located in arid or semi-arid regions, where the influence of salt stress is aggravated by the simultaneous action of xerothermic factors, especially high temperatures (Kuznestov and Shevyakova, 1997). Thus, the studies performed under controlled conditions in the laboratory through the application of a single stress do not adequately reflect the real-world conditions that occur in the field. In that respect, it has been previously shown that the transcript expression pattern in tobacco and Arabidopsis plants grown under a combination of drought and heat stress, or salinity and heat stress was different from that observed when these stresses were applied individually (Rizhsky et al., 2002, 2004; Mittler, 2006). An additional large-scale microarray experiment (Rasmussen et al., 2013), in which several abiotic and biotic stresses were studied in combination or as a single stress, concluded that about 60% of the total transcripts expressed could not be predicted from the corresponding single stress experiments alone. Our research group has also demonstrated that the synthesis and accumulation of some osmoprotectants was specifically altered under the combination of salinity and heat as compared to that observed under the heat or salt stresses applied individually (Rivero et al., 2014). These findings highlight the importance of studying abiotic stress combination for engineering or breeding for plant tolerance to real-world abiotic stress field conditions (Yamaguchi and Blumwald, 2005; Mittler, 2006).

Reactive oxygen species (ROS), such as the superoxide anion (${\text{O}}_{2}^{-}$), hydrogen peroxide (H_2_O_2_), and hydroxyl radical (HO), are formed in cells by the partial reduction of oxygen. Plants can be damaged by the accumulation of ROS not only on a daily basis, but also on a seasonal basis, as a consequence of their being subjected to long-term abiotic stress conditions, such as drought, salinity, low/high temperatures, or nutrient deprivation (Mittler, 2002). To mitigate the cytotoxic effects of ROS accumulation, plants have evolved antioxidant machinery consisting of enzymatic and non-enzymatic components. Among the enzymatic components, superoxide dismutase (SOD), ascorbate peroxidase (APX), glutathione reductase (GR), catalase (CAT), and peroxidases are the most important ones; whereas the most important non-enzymatic antioxidant compounds are glutathione, ascorbic acid, carotenoids, and flavonoids, among others. Their combined function (enzymatic and non-enzymatic) keeps the cell with an adequate balance of ROS (Rivero et al., 2001, 2007; Almeselmani et al., 2006). Phenylpropanoids have also been reported as antioxidant agents whose effectiveness depends on their reduction potential and accessibility within cells (Agati et al., 2012; Brunetti et al., 2013). Phenylpropanoids comprise a wide and important class of secondary metabolites, and have been suggested to play different and significant roles in plant responses to biotic and abiotic stresses, plant development, the maintenance of the integrity of plant structure, UV photoprotection, plant reproduction, and the internal regulation of plant cell physiology and signaling (Ferrer et al., 2008). Phenylpropanoids also serve as chemical modulators of plant communication with insects and microbes, with an active participation in defense-related phytoalexin responses to herbivory and infection, flower color for pollinator attraction, and the induction of root nodulation, among others. Among phenylpropanoids, flavonols have been recently described as powerful antioxidants (Agati et al., 2012; Brunetti et al., 2013). Recent evidence has shown that antioxidant flavonoids are located in the nucleus of mesophyll cells, chloroplast, and mitochondria, evidencing the flavonoids' important antioxidant activity, as they can accumulate at major targets of oxidative damage by ROS (Mursu et al., 2008; Modrianský and Gabrielová, 2009; Carrasco-Pozo et al., 2011; Lagoa et al., 2011; Visioli et al., 2011; Sandoval-Acuna et al., 2014). Severe stress conditions might inactivate antioxidant enzymes, while at the same time up-regulating the biosynthesis of some specific flavonols (Mittova et al., 2003; Modrianský and Gabrielová, 2009; Slimestad and Verheul, 2009; Sandoval-Acuna et al., 2014). It has been shown that flavonols have the ability to prevent ROS generation, but also quenching ROS once they are formed. Also, flavonols participate in the modulation of plant cell growth and differentiation, as well as the regulation of the activity of different protein kinases, which in turn are responsible for mediating ROS-induced signaling cascades that are vital for cell growth and differentiation (Agati et al., 2011, 2012; Brunetti et al., 2013). However, until now, the role that phenylpropanoids and/or flavonols could play under common abiotic stresses in nature, such as salinity or heat, still remain unclear. Agati et al. (2011) have suggested that flavonoids could constitute a secondary ROS-scavenging system in plants that are suffering from a severe excess of excitation energy being channeled to the photosynthetic apparatus due to salinity conditions, but these experiments were not conclusive.

Metabolomics is one of the most powerful emerging–omics approaches that can be used to peer into cellular biology, due to the vast amount of information that can be generated on the cell's metabolic state. In the past few years, metabolomic approaches have been critical for many biological studies. For example, for the functional identification of unknown genes (Bino et al., 2004; Hirai et al., 2005; Watanabe et al., 2008; Matsuda et al., 2010), the discovery of biomarkers associated with disease phenotypes (Soga et al., 2006; Sreekumar et al., 2009), the safety assessment of GMOs (Beale et al., 2009; Kusano et al., 2011), and the discovery of compounds involved in plant tolerance to biotic and abiotic stresses (Ward et al., 2010; Kusano et al., 2011). When combined with genomic, transcriptomic, and/or proteomic approaches, metabolomics can also help with the interpretation and understanding of many complex biological processes (Okazaki and Saito, 2012).

In this work, our main objective was to gain more insight into the changes that occur during ROS metabolism in tomato plants. Tomatoes are an economically-important crop world-wide, have many nutritional and health qualities, and are used as a model plant in many studies due to its many interesting features such as fleshy fruit, a sympodial shoot, and compound leaves, which other model plants (e.g., rice and *Arabidopsis*) do not have. The tomato plants in our study were grown under salinity, heat or the combination of both. The results obtained from transcript regulation and enzymatic activities did not fully explain the lower oxidative damage observed under the combination of salinity and heat as compared to salinity. Thus, we hypothesized that additional compounds might be involved in ROS detoxification during the different stresses studied. A metabolomics study performed under these conditions revealed that the type of phenolic compound that accumulated was very different depending on the stress applied, with flavonols accumulating in greater concentrations as compared to hydroxycinanmic acids. Moreover, the higher concentrations of flavonols correlated with lower oxidative damage, highlighting their role in cellular ROS homeostasis, the reduction of oxidative damage, and abiotic stress tolerance.

## Materials and methods

### Experimental design

Seeds of tomato plants (*Solanum lycopersicon* cv. Boludo, kindly provided by Monsanto) were sown in vermiculite in a growth chamber under control conditions of light (500 μmol m^−2^ s^−1^), photoperiod (16/8 h day/night), humidity (60–65%) and temperature (25°C) for 10 days until the appearance of the first two true leaves. A total of 78 plants were transferred to 18 L containers and grown in aerated hydroponic systems containing a modified Hoagland solution (Mestre et al., 2012) under the same environmental and control conditions described above. The pH of the nutrient solution was kept between 5.5 and 6.1 and the solution was renewed every 3 days. After 20 days of growing under control conditions, half of the plants (36 plants) were transferred to a growth chamber which was previously set at 35°C. Simultaneously, half of the plants in each growth chamber (18 plants) were treated with 80 mM of NaCl. Therefore, the experiment consisted of four treatments: control (25°C + 0 mM NaCl), salinity (25°C + 80 mM NaCl), heat (35°C + 0 mM NaCl), and salinity+heat (35°C + 80 mM NaCl). Plants were sampled 15 days after the treatments begun. All the plants were separated into roots, stems and leaves, and fresh weight (FW) was recorded. Half of the plants were dried in a forced air oven at 70°C for dry weight (Giovannucci, 1999) measurements. The other half was immediately stored at −80°C for further analyses. Whole tomato leaves from three different biological samples from each treatment applied were used for the analyses described below.

### RNA extraction and qRT-PCR experiments

Total RNA was isolated from whole tomato leaves from (as described above) using the TRI Reagent (Sigma-Aldrich, Ref. T9424). The RNA was cleaned with an RNAeasy spin column (Qiagen). To eliminate traces of DNA, the RNA was treated with DNase I (Fermentas Life Sciences) according to the manufacturer's protocol. cDNAs were synthesized from three separate RNAs (2 μg of total RNA) using the SuperScript VILO cDNA synthesis kit (Invitrogen). From each cDNA, three technical replicates were used, so that every sample was represented by nine replicates in total. Primer3 software was used for primer design based on the tomato unigenes sequences available at Sol Genomics Network (http://solgenomics.net/). The accession number of the genes assayed for expression and the primer sequence used are listed in Table S5 (for oxidative metabolism-related genes) and Table S6 (for phenolic metabolism-related genes). Two independent internal controls (actin and EIF-1α) whose expression did not change across different samples were used. A first normalization of the expression obtained for the different genes assayed in this experiment was done against actin. To ensure that actin was a real housekeeping gene, a second normalization was performed against EIF-1α, and no changes in the expression levels with respect to that obtained with actin normalization were observed. A total reaction volume of 20 μl was used. Reactions included 2 μl of template, 10 μl of Fast SYBR Green Master Mix, 0.9 μl of reverse primer, 0.9 μl of forward primer, and sterile molecular biology-grade water to a total volume of 20 μl. PCR assays were performed using the following conditions: 95°C for 10 min followed by 40 cycles of 95°C for 3 s and 60°C for 30 s. Melting curve analyses for all targets were carried out under the following conditions: 95°C for 15 s, 60°C for 1 min, and 95°C for 15 s. Amplification and data analysis were carried out using the ABI StepOne Plus real-time PCR system (Applied Biosystems) using actin and/or EIF-1α as the internal controls. The relative fold change (FC) was measured against the control plants samples. Log_2_ of the FC was calculated and represented in **Figure 5**. Relative expression values obtained from qPCR experiments can be found in Table S7 (for phenolic metabolism-related genes) and Table S8 (for oxidative metabolism-related genes).

### H_2_O_2_, lipid peroxidation and protein oxidation determination

#### H_2_O_2_ quantification

H_2_O_2_ was extracted as described by Macnevin and Urone (1953) with some modifications (Brennan and Frenkel, 1977; Rivero et al., 2007). The concentration of peroxide in the extracts was determined by comparing the absorbance against a standard curve representing a titanium–H_2_O_2_ complex from 0.1 to 1 mm.

#### Lipid peroxidation

Malondialdehyde (MDA), as a degradation product of lipid peroxidation, was determined as Fu and Huang (2001) with the modifications as listed in Mestre et al. (2012). The MDA concentration was calculated using an extinction coefficient for MDA of 155 mM^−1^cm^−1^.

#### Protein oxidation

Protein oxidation was assayed as according to Reznick and Packer (1994).

### Enzymatic activities

The extraction procedures for the enzymatic analyses were performed as in Li et al. (2013) with some modifications. For phenylalanine ammonia-lyase (PAL) and chalcone isomerase (CHI) the extraction was done on 0.5 g of frozen tissue. Tissues were homogenized at 4°C in 1 mL of 100 mM Tris–HCl buffer (pH 8.8) containing 14 mM β- mercaptoethanol, 5 mM DTT, 1% BSA, and 5% PVPP. For trans-cinnamate 4-monooxygenase (C4H) the same extraction buffer was used as for PAL but with pH 7.5. For chalcone synthase (CHS) and flavonone 3-hydroxylase (F3H), 0.5 g of frozen tissues were homogenized at 4°C with 5% PVPP in 1 mL of 100 mM sodium phosphate buffer (pH 6.8) containing 14 mM β-mercaptoethanol, 5 mM DTT, 40 mM sodium ascorbate, 3 mM EDTA, and 2% BSA at 0–4°C. The homogenate was centrifuged at 12,000 × *g* for 20 min at 4°C. The supernatant was precipitated with ammonium sulfate (70% w/v saturation), kept on ice for 1 h, and centrifuged at 16,000 × *g* for 10 min at 4°C. The resulting precipitate was dissolved in 1 mL of 100 mM sodium phosphate buffer (pH 6.8) containing 8 mM DTT, 40 mM sodium ascorbate, and 1% m/v BSA. After centrifugation, the enzyme extract was purified with ammonium sulfate. All the enzyme extracts were desalted with PD10 columns and then used for enzyme analysis immediately. Enzyme quantifications were performed spectrophotometrically using a temperature-controlled plate reader (Biotek PowerWave HT, Biotek Instruments, USA).

*PAL activity* was assayed in a mixture (250 μL) containing 100 mM Tris-HCl buffer pH 8.0 and enzyme extract. The reaction was initiated by the addition of 150 μL of 200 mg mL^−1^ L-phenylalanine (final concentration 6 mg mL^−1^) and the production of cinnamic acid was measured over 10 min at ΔA_290_.

*C4H activity* was assayed in a mixture (250 μL) containing 100 mM phosphate buffer (pH 7.5), 1 mM DTT, 1 mM NADPH, and 100 μL enzyme extract. The reaction was initiated by the addition of 10 mM trans-cinnamic acid (final concentration 1 mM) and the changes in absorbance at 290 nm was recorded during 10 min.

*F3H activity* was assayed in two independent reactions following the conversion of naringenin to dehydrokaempferol and the conversion of eridictyol to dihydroquercetin. The reaction mixture (250 μL) contained 100 mM Tris-HCl buffer (pH 7.5), 0.5 mM DTT, 0.25 mM 2-oxoglutarate, 0.05 mM ferrous sulfate, 5 mM sodium ascorbate, and 0.5 mM of the substrate (naringenin or eridictyol). The addition of the substrate initiated the reaction and the variation in absorbance at 280 nm was followed for 10 min. Results obtained from each substrate were combined and plotted as F3H activity.

*CHI activity* was assayed as follows: 2′,4,4′,6′-Tetrahydroxychalcone was synthesized from naringenin by treatment with 50% KOH followed by acidification and recrystallization with aqueous ethanol. Enzyme extract (100 μL) was added to 50 mM Tris-HCl pH 7.4 containing BSA (at a final concentration of 7.5 mg mL^−1^) and 50 mM KCN to give a final volume of 250 μL. The reaction was initiated by adding 5 μL of 1 mg mL^−1^ tetrahydroxychalcone in 2-ethoxyethanol and changes in absorbance at 381 nm was recorded with the cell holder maintained at 30°C. To allow for spontaneous isomerization of the chalcone, the reference cell contained the assay mixture without enzyme. The rate of disappearance of the chalcone (ΔA_381_), in the presence of enzyme, was used to estimate CHI activity.

*CHS and UFGT activities* were assayed as described by Ghasemzadeh et al. (2012) and Ju et al. (1995), respectively.

*DHAPS activity* was assayed as described in Wu et al. (2003).

*SHD activity* was assayed as shown in Tahmasebi-Enferadi et al. (2011).

*SK activity* was measured following Schmidt et al. (1990) with some modifications. The reaction mix consisted of 100 μL enzymatic extract, 50 mM buffer glycine-NaOH pH 10, 5 mM MgCl2, 5 mM DTT, 4 mM ATP. The reaction started by the addition of 0.33 mM shikimic acid and the absorbance was followed for 10 min at 290 nm.

*4CL activity* was assayed as Lee et al. (1997).

*C3H activity* was assayed by monitoring the production of 1,3-caffeoylquinic acid. The reaction mix consisted of 50 mM buffer Tris-HCl pH 7.4 containing 1 mM EDTA and 120 mM NADPH. The samples were incubated at 37°C for 3 min and the reaction was quenched by the addition of 0.1 mL of 25% aqueous trifluoroacetic acid. The absorbance was recorded at 350 nm by the disappearance of NADPH.

All the enzymatic activities were expressed as a function of the protein concentration of the extracts, which were assayed and calculated using the Bradford method (Bradford, 1976). All the enzymatic activities obtained were normalized against control samples and fold change (FC) of the normalized values were calculated. Log_2_ values were calculated from FC and shown in **Figure 5**. The absolute values for all the enzymes assayed can be found in Tables S9, S10.

### Metabolomics analysis and identification of metabolites

Six biological replicates of fresh tomato leaves for each treatment (control, salinity, heat and salinity, and heat) were used for metabolite profiling. The samples were prepared by extracting 1 g of fresh material with methanol:water (3:1, v/v). Methanol was the preferred solvent used as it was able to successfully extract most of the polar and semi-polar metabolites contained in tomato leaves (Gomez-Romero et al., 2010). Samples were analyzed using the Agilent 6550 UHPLC-QTOF-MS F as described by Tomás-Navarro et al. (2014). Initial processing of the raw data obtained was performed using the Agilent MassHunter Qualitative Analysis software v. 6.00 (Agilent Technologies; Figures S1, S2). Curated raw data obtained from UHPLC-QTOF-MS were subjected to a first level of analysis using XCMS (“*CentWave*” method, Smith et al., 2006). The results obtained by XCMS analysis were introduced in CAMERA, a Bioconductor package that integrates algorithms to extract compound spectra, annotate isotope and adducts peaks, and propose the accurate compound mass even in highly complex data (Kuhl et al., 2012). After CAMERA analysis (Table S1), the results obtained were subjected to a second level of analysis by normalizing against control samples and a statistical analysis consisting of a *t*-test and ANOVA was performed, so all the molecular features with a *P*_*adj*_ > 0.05 were eliminated from the study. These *P*_*adj*_ values were corrected by a Benjamini-Hochberg method. Then, fold-change and log_2_ of the fold-change of different molecular features found among treatments were calculated with respect to control plants, so all those with no significant statistical difference (log_2_ < 1 or >–1) were also eliminated from our analysis (Table S2, tabs “CvsS,” “CvsH,” “CvsSH”). The different molecular features remained after these cut-off analyses (those statistically different) were compared among the different treatments applied, with the objective of identify common and particular compounds for each treatment (Table S3, venn diagram **Figure 3B**).

Putative metabolite identification of the metabolites shared by all the stress conditions applied was performed using a two-stage approach: (1) A search based on the predicted elemental composition in the available databases (e.g., MOTO database (http://appliedbioinformatics.wur.nl/moto); KNApSAcK metabolite database (http://kanaya.naist.jp/KNApSAcK/); KOMOCS (Kazusa OMICS, http://webs2.kazusa.or.jp/komics/); MassBank (http://www.massbank.jp/); Madison 15 Metabolomics Consortium Database (http://mmcd.nmrfam.wisc.edu/); ARMeC: High Mass resolution annotation database (http://www.armec.org/MetaboliteLibrary/index.html); METLIN (https://metlin.scripps.edu/); Human Metabolome Database (HMBD: http://www.hmdb.ca/) within a mass tolerance of 10 ppm was performed. If no matched metabolites were found, the molecular feature was noted as unidentifiable, and only the matched metabolites were considered as candidates. 2) For each metabolite candidate, the spectrum coverage, the sum of the matched fragments intensities over the total fragment intensities in the spectrum was used to determine the underlying metabolite. If the metabolite could not be identified by spectrum match, we checked whether characteristics fragments existed in the different molecular features to reduce the number of candidates. Isotope ratio (IR) matches were also performed for all of them to ensure a metabolite's identity. Finally, retention times (RTs) from the different identified metabolites were checked again across the different databases, since they are usually relatively stable across experiments. Although similar RTs usually appear across samples, their relationship with a single mass almost always ensures them to be associated with a single metabolite. Finally, a comparison of the results obtained to those found in the literature (Moco et al., 2006; Iijima et al., 2008; Mintz-Oron et al., 2008; Yamanaka et al., 2008) was made and a final list of the identified metabolites is shown in Table S4.

### Analysis of phenolic compounds by HPLC/UV-PAD/ESI-MSn

For the identification and characterization of phenolics, 1 g of frozen plant material was extracted with 1 mL of water/methanol (20:80) by sonication for 1 h, followed by overnight extraction and another sonication period (1 h). The resulting extract was centrifuged and filtered through a 0.45 um PVDF membrane.

Chromatographic separations were carried out on a Phenomenex reverse-phase column [250 × 4.6 mm, Luna 5 um C18 (2) 100A]. The mobile phase consisted of two solvents: water/acetic acid (1%; A) and acetonitrile (B), starting with 5% B and using a gradient to obtain 50% at 30 min and 80% at 37 min. The flow rate was 1 mL/min and the injection volume 20 μL. Spectral data from all peaks were accumulated in the range of 200–400 nm, and chromatograms were recorded at 280, 320, and 360 nm. The HPLC/UV-PAD/ESI-MSn analyses were carried out with an Agilent HPLC 1100 series equipped with a Pulsed Amperometric Detector (Dorais et al., 2008) and mass spectrometer in series (Agilent Technologies, Waldbronn, Germany).

The mass spectrometer was an ion trap mass analyzer equipped with an electrospray ionization interface. The ionization conditions were adjusted to 350°C and 4 kV for capillary temperature and voltage, respectively. The nebulizer pressure and flow rate of nitrogen were 65.0 psi and 11 L/min, respectively. The full-scan mass covered the range from m/z 100 to 1200. Collision-induced fragmentation experiments were performed in the ion trap using helium as the collision gas, with voltage ramping cycles ranging from 0.3 to 2 V. Mass spectrometry data were acquired in the negative ionization mode. MSn was carried out on the most abundant fragment ion observed in the first-generation mass spectrum.

The identification of the peaks was obtained by analyzing the extracted-ion chromatograms of the ion current at m/z values corresponding to the [M–H]^−^ ions of the individual investigated compounds, as well as their fragmentation. Quantification of the identified compounds? was performed by HPLC-PDA detection using the external standard method with calibration graphs, as a function of concentration based on peak area, detected at the wavelength corresponding to their maximum absorbance.

### Statistical analysis

Statistical analysis for compound concentration was carried out as described above. Statistical analyses for DW, H_2_O_2_ concentration, MDA content, protein oxidation, and enzymatic activities were carried out with an analysis of variance (Giraud et al., 2008) using a *P* = *0.05* probability cut-off as indicative of significant differences followed by a post hoc pooled *t*-test [with significant values represented by ^***^P < 0.001, (Beale et al., 2009) *P* < 0.01, ^(*)^0.01 < *P* < 0.05] and a Duncan test when necessary. Relative transcript expression assayed by qPCR was calculated using the 2^−ΔCt^ method. Heat maps for metabolomics results, transcripts expression and enzymatic activities were created using R.

## Results

### Growth of tomato plants subjected to salinity, heat, and their combination

**Figure 1:**
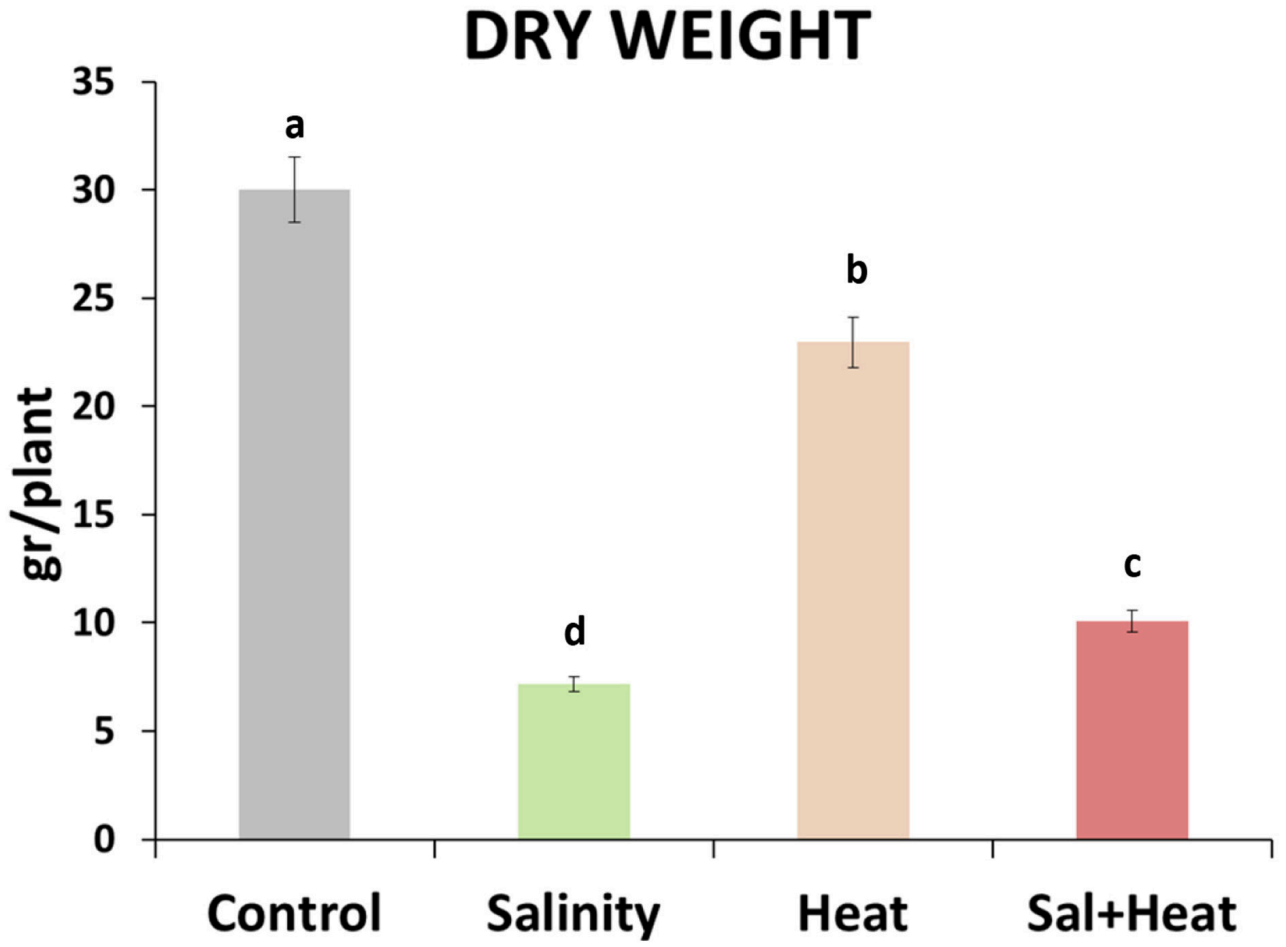
**Total dry weight of tomato plants grown under control conditions, salinity (80 mM NaCl), heat (35°C), or the combination of salinity and heat**. The figure is representative of two independent experiments. Values represent means ± *SE* (*n* = 12). Duncan test shows statistical differences among treatments (*P* < 0.05).

Tomato plants were grown under control (25°C, 0 mM NaCl) conditions for 25 days, and then either maintained under control conditions, or subjected to salinity (25°C, 80 mM NaCl), heat (35°C, 0 mM NaCl) or a combination of salinity and heat (35°C, 80 mM NaCl) for an additional 15 d (a whole vegetative-growth cycle). Dry weight of the plants was measured at the end of the experiment (Figure 1). Salinity, heat and the combination of salinity and heat caused a reduction in the plant's biomass, with salinity and salinity combined with heat treatments causing the most severe effects, resulting in reductions of 76 and 65%, respectively, with respect to control plants. On the other hand, plants grown under heat stress alone had a reduction of only 20% of their biomass, compared to control (Figure 1).

### Metabolism of reactive oxygen species in tomato plants subjected to salinity, heat, and the combination of salinity and heat

**Figure 2:**
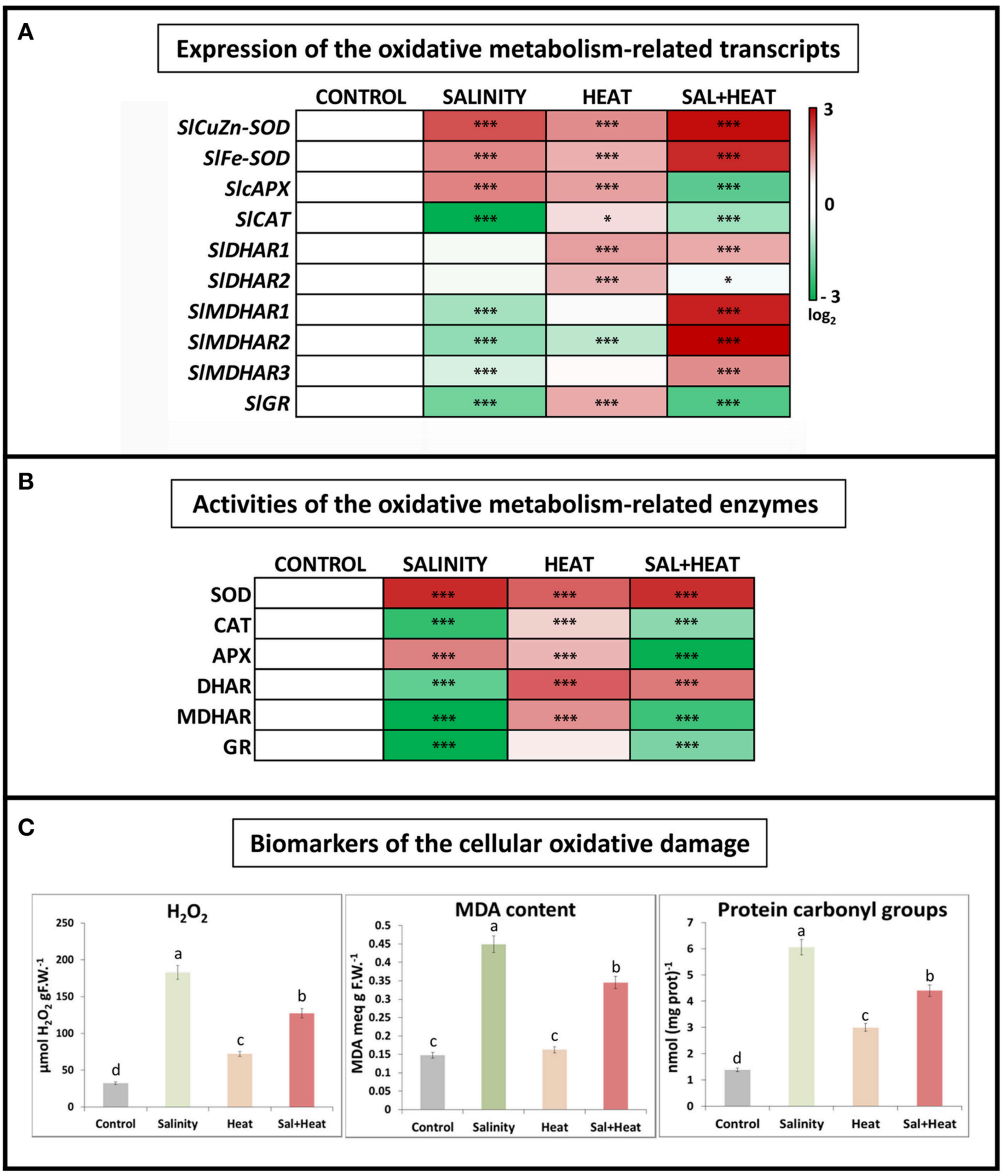
**Oxidative metabolism in tomato leaves grown under salinity, heat or the combination of salinity and heat**. **(A)**. Expression of oxidative stress metabolism-related transcripts. Scale is log_2_ of the mean values after normalization against control plants (*n* = 6). **(B)**. Activities of the ascorbate-glutathione cycle-related enzymes. Values are represented as the log_2_ of the absolute mean values after normalization against control plants (*n* = 6). **(C)**. Absolute measurements of the oxidative damage-related parameters (H_2_O_2_ concentration, MDA content, and Protein oxidation). Values represent means ± *SE* (*n* = 9). Duncan test shows statistical differences among treatments (*P* < 0.05). Asterisks mean statistically significant values respect to control at *P* < 0.05: ^***^P < 0.001, ^*^0.01 < *P* > 0.05, and no Asterisk means not significant differences.

The expression and activities of oxidative stress metabolism-related transcripts and enzymes were measured under salinity, heat, or their combination. As shown in Figure 2A, antioxidant-related transcripts displayed different expression patterns depending on the type of stress applied. Salinity resulted in a downregulation of most of the ascorbate-glutathione cycle enzymes, except for the SOD and APX transcripts which were upregulated, whereas heat led to an upregulation of most of the ROS-related transcripts measured (see Table S8). Interestingly, the combination of salinity and heat resulted in a specific pattern of transcript regulation, where CAT, APX, and GR were downregulated, and the rest of the transcripts analyzed were upregulated.

In addition to transcript expression, the activities of the main ascorbate-glutathione cycle enzymes were also measured. The results obtained (Figure 2B) showed a very similar pattern to that obtained after transcript profiling, with salinity being the treatment that displayed the largest inhibition of antioxidant enzyme activities, and heat being a general activator of the ascorbate-glutathione cycle enzymes. Under the combination of salinity and heat, some of these enzymes were activated (i.e., SOD, DHAR), whereas others (CAT, APX, MDHAR, and GR) were inhibited, as compared to control plants and similar to the salinity treatment.

Some of the biomarkers used to characterize the degree of oxidative damage experienced by plant cells under abiotic stress include hydrogen peroxide (H_2_O_2_) accumulation, peroxidation of membrane lipids (typically measured as the increase in MDA content) and protein oxidation (measured as protein carbonyl groups). These bioindicators were measured in tomato plants subjected to salinity, heat, and a combination of salinity and heat (Figure 2C). In response to all of the stresses applied, the levels of all three indicators increased significantly as compared to control plants. It is noteworthy to highlight that under heat stress, the concentrations of H_2_O_2_, MDA, and protein carbonylation were markedly lower than those under salinity and heat combination, as well as salinity alone. On the other hand, a negative and significant correlation was found between the expressions of the antioxidant-related transcripts studied and the content of the oxidative stress bioindicators, such that when the antioxidant transcripts were downregulated, the levels of H_2_O_2_, protein oxidation and MDA increased, and vice versa (Figure 2C).

The results obtained from the ascorbate-glutathione cycle transcript expression and enzymatic activities did not fully correspond with the results obtained for the biomarkers of cellular oxidation measured under the combination of salinity and heat. Therefore, although the inhibition of the ascorbate-glutathione cycle-related enzymes observed under salinity and the combination of salinity and heat was similar, the concentration of the oxidative damage biomarkers were much higher under salinity (Figure 2C). To determine whether this difference was due to the accumulation of alternative compounds or pathways that could potentially detoxify ROS, we conducted a detailed metabolomics study of tomato plants that were subjected to salinity, heat, and their combination.

### Metabolomics study of tomato plants subjected to salinity, heat, and a combination of salinity and heat

**Figure 3:**
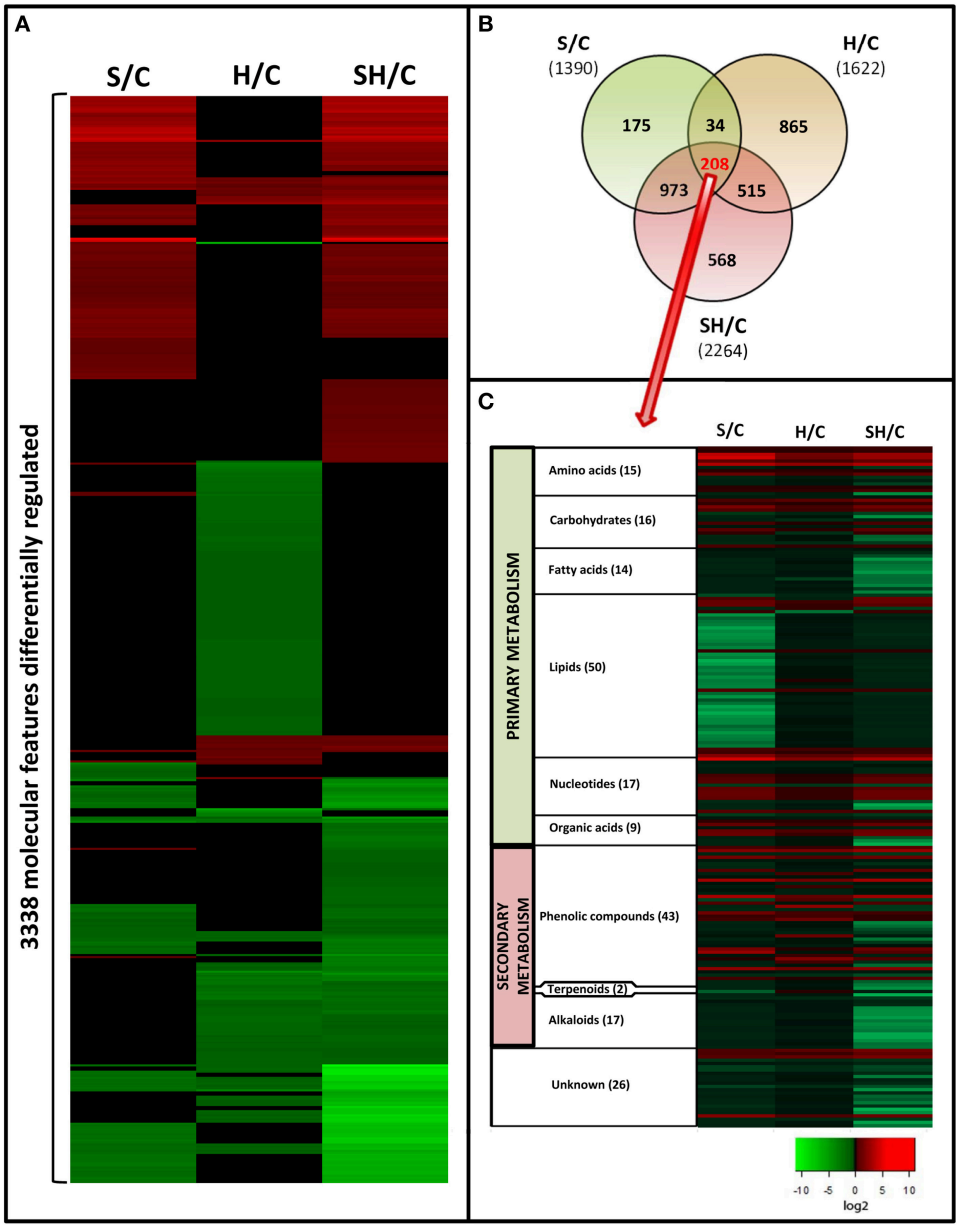
**Metabolomics study in tomato plants grown under salinity, heat or the combination of salinity and heat**. **(A)**. Changes in the accumulation of the different compounds that passed statistical significant tests (see Materials and Methods Section). Green means “down-regulation,” red “up-regulation,” and black “not differentially regulated.” **(B)**. Venn diagram of the significantly altered compounds under the different treatments applied. **(C)**: Classification and changes in accumulation of the molecular features (208) common to all the treatments applied.

Tomato leaf extracts from plants grown under salinity, heat, or a combination of salinity and heat were analyzed with UPLC-QTOF-MS (Figure S1). From all the acquired metabolites and after applying a *q*-value of *p*<*0.05* and log_2_ cutoffs of < –1 or >1, 3338 molecular features were found to be significantly different as compared to control plants under the three treatments applied. The different fold change log_2_ values of specific molecular features as compared to control samples are shown as a heat map (Figure 3A). Green indicates down regulation, red indicates up regulation, and black denotes molecular features that did not show significant differences with respect to the control treatment. It is remarkable that the combination of heat and salinity had a metabolomic pattern that could not be deduced from the results of the two different stresses applied singly. From the 3338 significantly-changed metabolites, a Venn diagram was created (Figure 3B and Table S3), which revealed the following information: 208 compounds were commonly altered by all three conditions applied. Interestingly, each of the treatments applied had a specific metabolic signature, i.e., some compounds that only changed in just one of the treatment conditions and not with the other two. Thus, 175 metabolites were specifically altered when salinity was applied as the sole stress, and 865 were specifically altered in response to heat. Curiously, the combination of salinity and heat specifically altered the level of 568 compounds that did not change under salinity or heat applied individually. These 568 compounds may represent the unique response of tomato plants to stress combination.

To increase our knowledge on the different pathways that were affected in response to all three different stress conditions, we focused on the 208 compounds that were similarly altered in response to all three stresses. Retention time, as an indicator of polarity, allows for predicting the class of metabolite being separated, according to the region where each family of metabolites elutes during chromatographic separation (Gomez-Romero et al., 2010). Based on this, the 208 molecular features shared by all the stress conditions used in our experiments were clustered into compound families (Figure 3C). From these, 26 molecular features could not be classified (unknown, Figure 3C).

Among the different families of compounds found to be differentially altered under all of the stresses applied, lipids (50 compounds identified) and phenolic compounds (43 compounds identified) were the most represented groups of compounds in our tomato plants. Very little is known about how phenolic compounds are regulated during salinity or heat stress, and information is even more scarce for the combination of salinity and heat. Therefore, we decided to deepen our understanding of these compounds and their metabolism under the different stress scenarios investigated.

### Metabolism of phenolic compounds in tomato plants grown under salinity, heat, or a combination of salinity and heat

As shown in Table 1, the metabolomics study revealed that heat stress induced the accumulation of kaempferol 3-O-glucoside, naringenin, naringenin chalcone, quercetin-3-hexoside, whereas these compounds were down-regulated (as compared to control) under salinity or the combination of salinity and heat. On the other hand, 1,3-dicaffeoylquinic acid, 1-feruoyl-5-caffeoylquinic acid, and 3-caffeoyl-1-5 quinolactone were specifically downregulated under heat, but accumulated under salinity or the salinity and heat combination. Furthermore, kaempferol, quercetin-3-rutenoside (rutin), and dihydrokaempferol accumulated under heat and the combination of salinity and heat, but not under salinity alone (Table 1). As phenolics have been defined as powerful antioxidant molecules in many publications, the decision was made to further identify/quantify these molecules in order to understand the regulatory processes that govern their biosynthetic pathways.

**Table 1:** **Phenylpropanoid metabolism-related compounds found among the differentially expressed metabolites in tomato leaves grown under salinity, heat or the combination of salinity and heat**.

**Compound name**	**mz**	**rt**	**C vs. S**	**C vs. H**	**C vs. SH**	**Adduct**	**Compound mass**	**HMDB ID**
***1,3-Dicaffeoylquinic acid***	551.0977	48.54	4.23656615	−1.23652119	2.38811887	M+Cl-	516.12677	HMDB29279
***1-Feruloyl-5-caffeoylquinic acid***	264.0635	52.61	2.35733238	−1.52105591	2.72545238	M-2H-	530.1424	HMDB41643
***3-Caffeoyl-1,5-quinolactone***	335.0788	79.785	2.25988935	−1.21884902	2.17358815	M-H-	336.08451	HMDB29287
3-Hydroxyflavone	316.9811	54.225	4.6706772	2.65337539	4.37004688	M+Br	238.062994	HMDB31816
5-O-Digalloyl-3,4-di-O-galloylquinic acid	913.1191	48.5	−1.98939261	−1.06902479	−5.42110942	M+TFA-H	800.107223	HMDB39345
***Cinnamic acid***	147.0453	55.41	3.58395655	3.31282643	4.14821129	M-H-	148.05243	HMDB00567
Cyanidin 3-O-dimalonyl-laminaribioside	804.1242	67.18	4.01701266	3.1605697	3.75559475	M+Na-2H	783.161998	HMDB29242
***Dihydroquercetin***	303.0521	56.345	−1.12656037	−1.79165259	−1.22674848	M-H-	304.0583	HMDB41418
Fukiic acid	308.9968	57.34	−1.81897922	−1.79795656	−1.1575458	M+K-2H	272.053217	HMDB29496
Isopeonidin 3-glucoside	542.0338	51.83	3.23575101	1.05855778	2.93822541	M+Br	463.124037	HMDB41752
***Kaempferol***	321.0171	66.19	−3.6943988	2.25979761	1.03060996	M-2H-	286.04773	HMDB05801
Kaempferol 3-sophoroside 7-glucuronide	821.1459	65.32	−2.44551444	−2.70383468	−4.20795587	M+Cl	786.185473	HMDB38769
***Kaempferol 7-neohesperidoside***	629.1273	53.625	−1.79632193	1.93633998	−1.02395371	M+Cl-	594.15847	HMDB37573
***(Kaempferol 3-o-glucanoside)***								
***L-Phenylalanine***	164.0718	55.12	5.23654856	3.12548658	4.25631579	M-H-	165.078979	HMDB00159
Luteolin 7-sulfate	364.9929	57.03	1.60632611	1.87477701	1.25233792	M-H	366.004553	HMDB38472
Luteolin 7-sulfate	346.9906	53.12	3.14739844	1.94111534	1.83369465	M-H20-H	366.004553	HMDB38472
Luteolinidin	308.0009	56.09	−1.13893716	−1.7086559	−1.1211963	M+K-2H	271.060648	HMDB29249
***Naringenin***	579.1720	66.505	−1.2428317	1.61778675	−1.0619038	M-H-	272.068473	HMDB02670
***Naringenin Chalcone***	307.0376	558.74	−1.6143945	1.67290633	−1.11862467	M+Cl-	272.06847	HMDB29631
***p-coumaric acid***	199.0167	59.23	3.2125256	2.32365623	3.01254585	M+Cl	164.047345	HMDB30677
p-Coumaroyl vitisin A	820.1469	64.99	−1.24899909	−1.46317044	−1.53201967	M+TFA-H	707.16121	HMDB29239
***p-Coumaroyl-CoA***	912.1448	48.51	1.84140384	1.04667792	2.30127143	M-H-	913.15198	HMDB40473
***Quercetin 3-β-D-glucoside***	463.0898	65.72	−1.08370176	2.99060206	−1.65827646	M-H-	464.0954761	HMDB37932
***(quercetin 3-hexoside)***								
Quercetin 4′-glucoside	445.0732	65.19	−1.57929402	−1.83228318	−3.19741223	M-H20-H	464.095476	HMDB37932
***Rutin (quercetin-3-rutenoside)***	609.1464	53.79	1.0235684	3.53021724	2.26787098	M-H-	610.15338	HMDB03249
***Dihydrokaempferol***	287.0656	52.39	−1.90584957	1.98437631	1.14502105	M-H-	288.06338	HMDB30847
6alpha-Hydroxyphaseollin	337.1137	217.98	−2.22025818	−2.16096822	−3.3566615	M-H	338.115424	HMDB30771
Malvidin 3-galactoside	538.1117	203.25	−1.27324403	−1.64393787	−1.02814672	M+FA-H	493.134601	HMDB38010
***Quercetin***	337.0114	244.91	−2.10044283	3.9941919	1.11021215	M+Cl-	302.04265	HMDB05794
[4]-Gingerdiol 3,5-diacetate	1055.5415	48.555	−2.10126613	−1.26872032	−6.99923873	3M-H	352.188589	HMDB39132
1-(Isothiocyanatomethyl)-4-methoxybenzene	215.9827	51.87	1.99979221	1.48139272	2.49735486	M+K-2H	179.040485	HMDB32581
2,4-Dibromophenol	248.8529	54.38	−2.45969985	−1.0611777	−2.62773254	M-H	249.86289	HMDB32079
3-Iodothyronamine	433.9262	84.41	−1.97622117	−1.49141479	−4.33072751	M+Br	355.006922	HMDB60524
5-Sulfo-1,3-benzenedicarboxylic acid	290.9822	73.575	−1.1732639	−1.10998653	−1.34366862	M+FA-H	245.983423	HMDB32822
Tyramine-O-sulfate	295.9550	47.89	4.39619601	3.14371076	4.51180665	M+Br	217.040879	HMDB06409
Dichlorphenamide	382.8420	48.51	−2.00096043	−1.14313963	−7.59097017	M+Br	382.833488	HMDB15275
Hexachlorophene	384.8411	48.51	−1.90345108	−1.07168251	−6.37842871	M-H20-H	403.849896	HMDB14894
Scopolamine	382.0678	55.83	1.873181	3.00076128	1.33061041	M+Br	303.147058	HMDB03573

*Red boxes show up-regulated compounds and green boxes show down-regulated compounds compared to control treatment. Values are expressed as log_2_ of the normalized values against control plants. Values represent means ± SE (n = 6)*.

**Figure 4:**
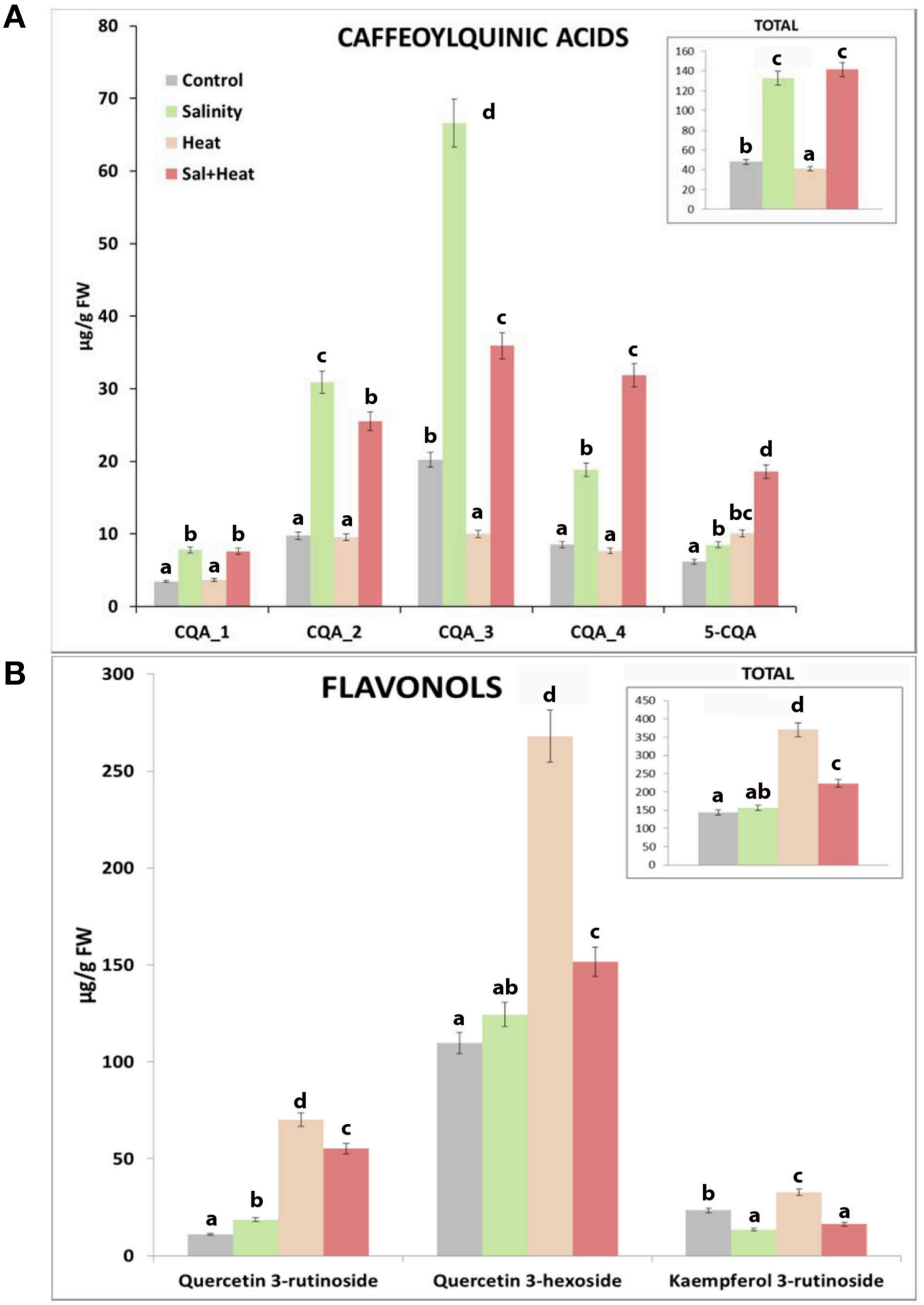
**HPLC analysis and quantification of some phenylpropanoid compounds in tomato plants grown under salinity, heat or the combination of salinity and heat**. **(A)**. Caffeoylquinic acids concentration. **(B)**. Flavonols concentration. Values represent means ± *SE* (*n* = 9). Duncan test shows statistical differences among treatments (*P* < 0.05).

We thus performed a HPLC quantification of the caffeoylquinic acids and flavonols present in our tomato samples under the different stress conditions used (Figure 4) in order to confirm the results obtained from the metabolomics profile. The HLPC analysis showed 5 well-differentiated peaks corresponding to Caffeoylquinic acids (Figure S2, C1–C5) and 3 other peaks related to flavonols (Figure S2, F1–F3). The data indicated that caffeoylquinic acids had a similar behavior in both control and heat-stressed plants, with much reduced levels (3.5-fold less) than those found under salinity or the combination of salinity and heat (Figure 4A). However, the concentration of flavonols was much higher under heat stress than under salinity or the combination of salinity and heat (Figure 4B). Under salinity, the concentration of flavonols was similar to control plants. Curiously, under the combination of salinity and heat stress, the concentrations of some flavonols (quercetin-3-rutenoside and quercetin-3-hexodide) were greater than those found under control and salinity stress applied singly, but were always lower than the concentration of these compounds under heat stress (Figure 4B).

**Figure 5:**
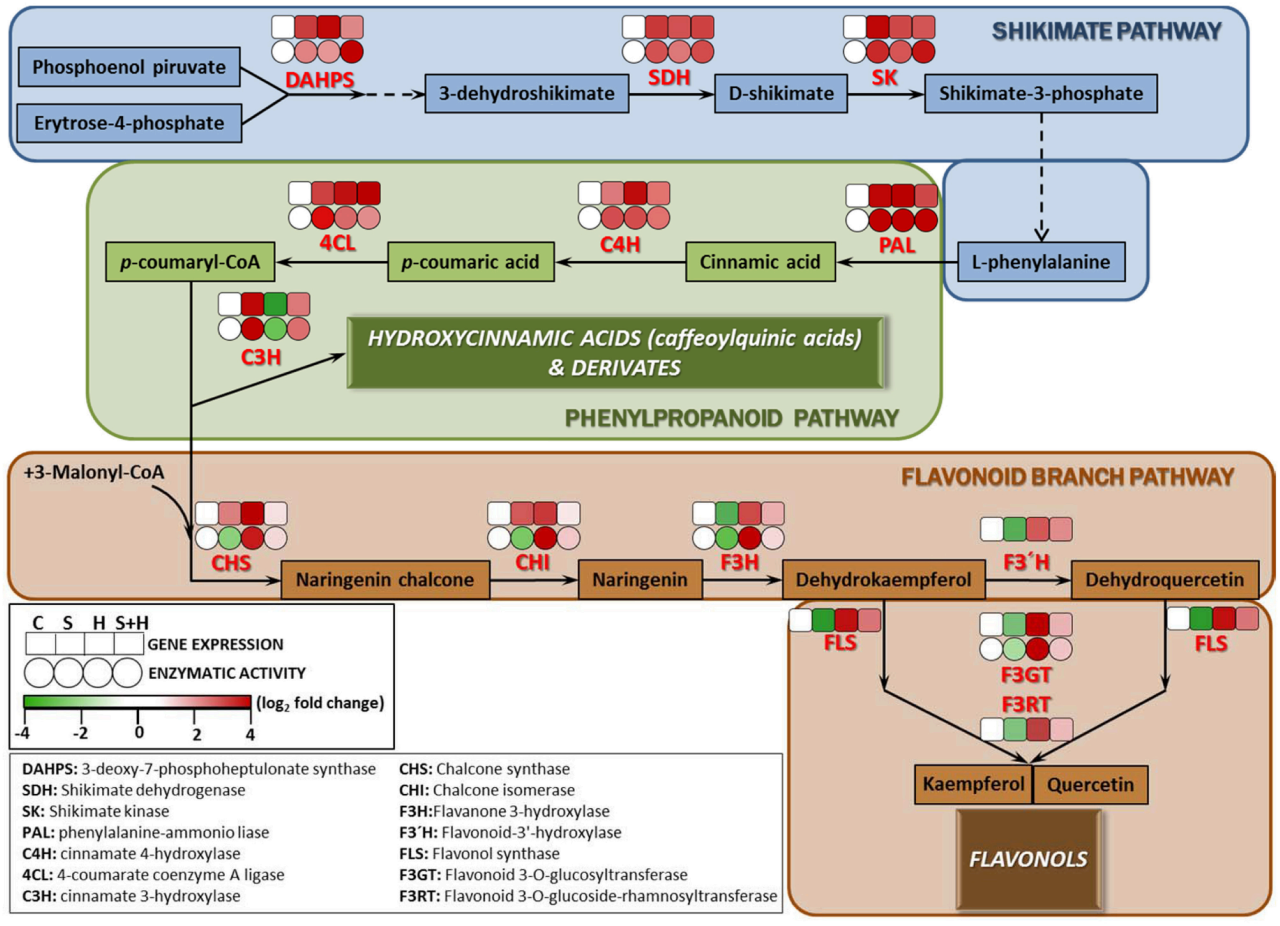
**Scheme of the proposed changes in phenolic metabolism in tomato plants subjected to heat, salinity and a combination of heat and salinity**. Differences in the activities (circles) of the enzymes involved in the phenylpropanoid pathway, and the expression (squares) of the transcripts that code for these enzymes under salinity, heat, and the combination of salinity and heat compared to control conditions are shown. A red color represents up-regulation, whereas green color shows down-regulation respect to the control treatment. Scale is the log_2_ of the mean (expression or activity) values after normalization against control plants (*n* = 6). Absolute activity values as well as log_2_ values of the enzymatic activities and transcript expression can be found in Tables S7–S9, respectively.

Due to the differences found among the phenolic compounds accumulating under the different stresses applied (Figure 4), a detailed study at transcript and enzyme levels was carried out in order to acquire new insights on how their biosynthetic pathways were regulated under the different stresses (Figure 5). In this sense, the activity of the main enzymes involved in phenylpropanoid metabolism, as well as the expression of the transcripts that coded for these enzymes were studied. Figure 5 shows a schematic view of phenylpropanoid metabolism in tomato plants. Enzymatic activities are represented as circles and transcript expression as squares. The green color shows up-regulation and the yellow color shows down-regulation of activity and/or expression, with the white denoting non-significant changes with respect to control conditions. As shown, synthesis of hydroxycinnamic acids was up-regulated at both the transcript expression and enzymatic activity levels under every stress condition applied (salinity, heat, or the combination of salinity and heat) (Figure 5, Shikimate and Phenylpropanoid pathways). However, the last limiting step in the hydroxycinnamic acids synthesis, cinnamate 3-hydroxylase (C3H) was down-regulated under heat stress at both transcript expression and enzymatic activity levels. A positive correlation between the inhibition of gene expression and enzymatic activity and a low concentration in caffeoylquinic acids under heat stress was also observed (*SlC3H*-caffeoylquinin acids concentration *r* = 0.854^***^; CH3 activity-caffeoylquinic acids concentration *r* = 0.902^***^). On the other hand, it was observed that transcript expression of CHS and CHI, the main branch enzymes of flavonol synthesis (Figure 5, Flavonoid branch pathway), were upregulated under all of the stresses applied, but their corresponding enzymatic activities were inhibited when salinity was applied as a sole stress, indicating a post-transcriptional inhibition of the process. F3H, F3′H FLS, F3GT, and F3RT showed, on the other hand, an inhibition at transcript expression and enzymatic activity levels under salinity (Figure 5, Flavonoid branch pathway), whereas under the combination of salinity and heat this inhibition was not so evident, and their values were similar to those observed under control conditions.

**Figure 6:**
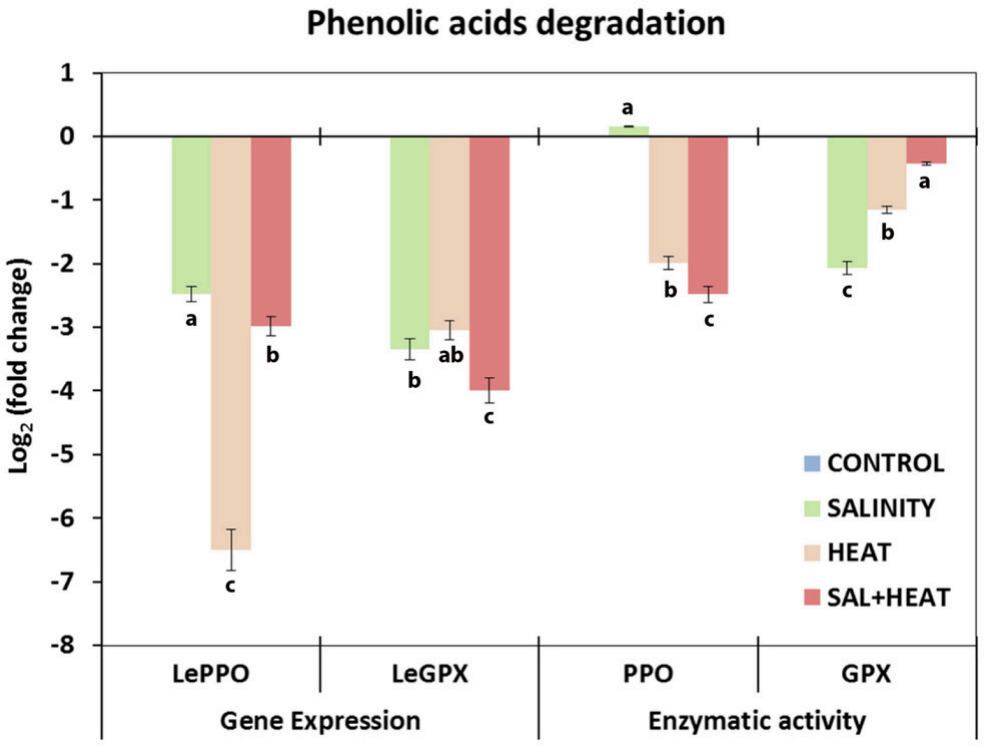
**Activity of the main enzymes involved in phenolic acids degradation and expression of the transcripts that code for these enzymes in tomato leaves**. Scale is the log_2_ of the mean (expression or activity) values after normalization against control plants (*n* = 6). Absolute activity values as well as log_2_ values of the enzymatic activities and transcript expression can be found in Supporting Information Tables S8, S9. Duncan test shows statistical differences among treatments (*P* < 0.05).

To complete the phenylpropanoid pathway study, transcript expression and activities of the main enzymes (PPO and GPX) involved in phenylpropanoid degradation were analyzed under the different conditions used in our experiment. As shown in Figure 6, both transcript expression and activities of PPO and GPX were markedly inhibited under all of the conditions applied, which was in agreement with the accumulation of phenolic compounds under these conditions. A negative and highly significant relationship between the accumulation of these compounds and transcript expression and/or enzymatic activities of the transcript/enzymes involved in the degradation of phenolic compounds was found (caffeoylquinic acids-*SlPPO r* = –0.896^***^; Flavonols-*SlPPO r* = –0.902^***^; caffeoylquinic acids-*SlGPX r* = –0.812^***^; Flavonols-*SlGPX r* = –0.884^***^).

## Discussion

To date, most molecular studies performed on tomato and other horticultural plants of economic interest have been performed via the application of a single stress condition. However, plants in nature are usually exposed to a combination of two or more stresses at the same time, and laboratory experiments performed on plants subjected to a single stress condition do not appropriately address the metabolic changes that plants suffer under conditions found in the field (Suzuki et al., 2014). Salinity and heat represent two important abiotic stresses for agricultural production as they cause important production losses and lead to reductions in fruit quality of horticultural plants. Moreover, the effects of climate change are being felt as a progressive increase of the salinity of irrigation water (low-quality water) and in the increase in the average temperatures worldwide. Our previous research has shown that the response of tomato plants to salinity and heat combination, during the first 72 h, was different from that observed when these stresses were applied individually, through the specific regulation of osmoprotectant accumulation under these conditions (Rivero et al., 2014). These studies highlight the importance of studying abiotic stresses combinations.

In the present work, we aimed to study how oxidative metabolism and other related antioxidants were modified during a 15-days exposure of tomato plants to stress combination, using a metabolomics approach backed by the use of biochemical and molecular tools. The first interesting result was that plants grown under the combination of salinity and heat had a greater production of biomass that those grown under salinity alone. We observed that salinity applied as a sole stress reduced the tomato plants' dry weight by about 76% (the highest reduction) while the combination of salinity and heat reduced it by about 66%, compared to control plants (Figure 1). In contrast, heat stress applied individually only reduced it to about 20% with respect to control plants. These results were similar to those shown in our previous work, in which these stresses were applied for just 72 h (Rivero et al., 2014) and heat stress did not induce a large reduction in growth as compared to control plants. However, salinity resulted in the most aggressive treatment with the lowest biomass production, significantly lower than even the combination of salinity and heat. In our previous work, we also reported that the accumulation of glycine betaine and trehalose observed after treatment with a combination of salinity and heat could help maintain a high K^+^ uptake and thus a low Na^+^/K^+^ rate, whereas under salinity alone, an inhibition of K^+^ uptake was clearly observed.

### An oxidative stress-specific signature observed for the different abiotic stresses applied

Abiotic stresses cause oxidative damage to plant cells (Mittler, 2006). An impairment of photosynthesis under abiotic stress could, for example, originate via an excess of electrons that react with O_2_, leading to ROS production (Ahuja et al., 2010; Miller et al., 2010a). Our analyses of the degree of cellular damage caused by ROS (measured as an increase in H_2_O_2_, lipid peroxidation and protein oxidation levels) revealed similar levels to those obtained in previous experiments (Rivero et al., 2014). Membrane peroxidation, lipids, and the degradation/oxidation of proteins and DNA damage are among the most researched consequences of ROS action on cellular structures and are found under any kind of abiotic or biotic stress conditions (Blokhina et al., 2003; Davies, 2005; Foyer and Noctor, 2008; Rinalducci et al., 2008; Foyer and Shigeoka, 2011). Our results showed that salinity, heat, and the combination of salinity and heat induced an overproduction of H_2_O_2_, a high level of MDA (considered to be the main indicator of lipid peroxidation) and high levels of protein oxidation, with salinity applied as a sole stress being the most devastating in that sense. The combination of salinity and heat also induced high levels of lipid peroxidation and protein oxidation as compared to control plants, but at lower levels than salinity alone.

To mitigate the over accumulation of ROS, plants have evolved multiple ROS scavenging mechanisms. These include both enzymatic and non-enzymatic scavenging systems. Within the enzymatic scavenging system, the group of redox enzymes comprising the ascorbate-glutathione cycle are essential, and they were studied in depth, at the transcript expression and enzymatic activity levels. Our results suggested that the overproduction of H_2_O_2_ under the different stresses was due to an overexpression of *SISOD*-encoding transcripts (Figure 2A) and a concomitant increase in SOD activity (Figure 2C). H_2_O_2_ could be detoxified to H_2_O and O_2_ through CAT activity, or through the ascorbate-glutathione cycle thanks to the activity of APX (Mittler, 2002). Salinity and salinity combined with heat resulted in downregulation of the *SlCAT* transcript (Figure 2A), leading to the inhibition of CAT enzymatic activity as well (Figure 2B). This may explain the over accumulation of H_2_O_2_, as well as the degree of lipid peroxidation and protein oxidation observed under these two types of stresses by the overproduction of ROS (Figure 2C). In contrast, when salinity was applied as a single stress, a downregulation of all the transcripts belonging to the ascorbate-glutathione cycle, except for APX, was observed. It is well known that salinity can result in the inhibition of the plant's antioxidant systems and these observations have been previously reported for several plant species (Serrato et al., 2004; Borsani et al., 2005; Abbasi et al., 2007; Zhu et al., 2007; Giraud et al., 2008; Miller et al., 2010a; Mestre et al., 2012; Rivero et al., 2014). Furthermore, environmental stresses, such as salinity, have been shown to perturb the redox state, inhibit antioxidant mechanisms and increase ROS production in cells (del Rio et al., 1996; Mittova et al., 2003; Nyathi and Baker, 2006; Zhu et al., 2007). In tomato, salinity decreases ascorbate and GSH contents and induces lipid peroxidation in peroxisomes (Mittova et al., 2003), which is in agreement with our results.

Contrary to salinity, fewer studies have focused on how a high-temperature environment can affect the plant's antioxidant system and other plant metabolic pathways. Heat stress significantly impacts normal physiological processes such as photosynthetic dark respiration, membrane stability and mitochondrial respiration (Almeselmani et al., 2006), with these alterations accompanied by the formation of ROS (Evers et al., 2010). The tolerance of crop plants to high temperature stress has been reported to be associated with an increase in antioxidant enzymes activity (Sairam et al., 2000). In our experiment, heat stress increased the expression of all the antioxidant-related transcripts, except for *SlMDHAR2* (Figure 2A); however, values of MDHAR activity were very high (Figure 2B), which may indicate that the main isoenzyme in tomato plants is not only *SlMDHAR2* but others as well. These results may explain why the level of H_2_O_2_, lipid peroxidation and protein oxidation found under heat stress were the lowest found under any of the treatment conditions applied in our experiment (Figure 2). Interestingly, we observed that the expression of the antioxidant-related transcripts under the combination of salinity and heat behaved differently than that described previously under salinity or heat alone (Figure 2A). In the treatment where salinity and heat were combined, *SlCAT, SlAPX*, and *SlGR* were downregulated (as well as their CAT, APX, and GR enzymatic activities). The downregulation of the *SlGR* transcript may be related to the high levels of lipid peroxidation and protein oxidation found in plants grown under salinity and heat combination as compared to control plants, as described by Ursini et al. (1997). However, the salinity and heat combination inhibited the ascorbate-glutathione cycle (at both transcript and enzymatic levels, Figures 2A,B), a finding that did not explain the lower H_2_O_2_, lipid peroxidation and protein oxidation levels found under the stress combination.

Because ROS are produced in different cellular compartments, and because different ROS differ in their properties such as diffusion through biological barriers, solubility, and propensity to react with various biological molecules, plants have developed additional scavenging mechanisms that function in parallel to the enzymatic systems already described above. Examples of such parallel ROS detoxification/prevention mechanisms include antioxidant compounds that have the ability to scavenge ROS efficiently, such as β-carotene, anthocyanins, α-tocopherol, and phenolic compounds (Mittler, 2002; Mittler et al., 2004; Ahuja et al., 2010; Miller et al., 2010b). To identify additional ROS scavenging mechanisms that could mediate tolerance to the different stresses studied, we undertook a comprehensive and un-biased metabolomic study, with the aim of identifying the potential antioxidant molecule(s) that could be working in parallel to the ascorbate-glutathione machinery under the different stresses studied.

### Differences in the type of phenolic compounds accumulated and their biosynthetic pathways between the different stress treatments

We conducted an exhaustive analysis and putatively identified the most important compounds present in all the treatments applied in this experiment. We decided to focus on the characterization of the compounds that were commonly altered under all of the conditions used in our experiments, such as a single abiotic stress or stresses applied in combination. Thus, the information gathered could be extrapolated to different scenarios in the different agricultural areas of the world. From all the compounds shared by all the conditions applied, 208 molecular features were identified and classified as putative compounds. Our results showed that these compounds belonged to the main families of primary and secondary metabolic pathways. Lipids were found to be the major group of compounds that were modified under all of the stress conditions studied. As previously shown, and in agreement with our results (Figure 2C), these molecules were the main targets for oxidative modifications (Anjum et al., 2015), and under the three conditions applied these compounds showed a similar downregulation pattern (Figure 3C), with this downregulation being lesser under heat, and higher under salinity. The metabolomics study performed in our experiment also revealed that the second major group of differentially-regulated compounds was the phenolic metabolism-related compound group (Figure 3C). Interestingly, and contrary to what was observed with lipids, in this case the type of stress applied resulted in a different type of regulation of most of these compounds. As shown in Table 1, it was noted that under heat stress, seven of the phenylpropanoid-related compounds listed showed an opposite regulation than under salinity or salinity combined with heat, while three of them had a similar regulation pattern after the salinity and heat treatments. Curiously, from these compounds, the ones belonging to the caffeoylquinic acids family were down-regulated under heat, whereas the same compounds were up-regulated under salinity or the combination of salinity and heat. In contrast, compounds belonging to the flavonol family were up-regulated under heat, whereas under salinity and salinity combined with heat these compounds were down-regulated. The quantification of these compounds with HPLC corroborated these observations, with caffeoylquinic acids preferentially accumulating under salinity and salinity and heat combination, whereas flavonols accumulated under heat, although a significant increase of these compounds was also observed under the salinity and heat combination. Since the DW of tomato plants grown under heat stress was higher than those grown under salinity or the combination of salinity and heat, the decision was made to study the phenylpropanoid pathway (at the gene, enzymatic activity, and compounds levels) under the different scenarios used in our experiments.

The enzyme 3-deoxy-D-arabino-heptulosonate (DAHPS) is a key determinant of the flow of carbon into the shikimate pathway (Zhang et al., 2015). This enzyme was highly upregulated under all stresses applied in our experiments (Figure 5, Shikimate pathway). The shikimate pathway generates L-phenylalanine which is the main amino acid for the synthesis of hydroxycinnamic acids. The other main enzymes related to the shikimate pathway (SHD, SK), as well as the expression of the transcripts that encode for these enzymes were also upregulated under all the conditions used in our experiments (salinity, heat, and the combination of salinity and heat). This is also in agreement with the levels of L-phenylalanine encountered in our tomato plants under these stresses (Table 1), which were always several folds higher than the level of this amino acid in control plants. After exiting the Shikimate pathway, L-phenylalanine is then converted into cinnamic acid and redirected into the synthesis of the different phenolic compounds (Figure 5, phenylpropanoid pathway). From L-phenylalanine and through the participation of several important enzymes, such as PAL, C4H, and 4CL, the synthesis of *p*-coumaryl-CoA (the activated intermediate for hydroxycinnamic acids and their derivatives, and flavonols and flavonol derivatives) occurs (Besseau et al., 2007; Ferrer et al., 2008). The activity of these enzymes were positively correlated with the concentration of the intermediate compounds (cinnamic acid, p-coumaric acid and p-coumaryl-CoA), which were several folds higher following the salinity, heat and salinity combined with heat treatments, compared to those found in tomato plants grown under control conditions (Figure 5, phenylpropanoid pathway). Therefore, and up to this point in the phenolic pathway, all the stresses studied had a similar behavior, overproducing p-coumaryl-CoA, and its intermediates. p-coumaryl-CoA may also be used as the substrate for the synthesis of hydroxycinnamic acids (phenylpropanoid branched pathway) or flavonols (flavonols branched pathway). C3H is the last key limiting enzyme for the synthesis of hydroxycinnamic acids and derivatives from p-coumaryl-CoA (Ferrer et al., 2008). In this sense, it was observed that heat stress inhibited C3H at the transcript expression and enzymatic activity levels (Figure 5, phenylpropanoid pathway), which was in agreement with the concentration of hydroxycinnamic acids found in our analysis, being significantly lower than control plants (Figure 4, Table 1: 1,3-dicaffeoylquinic acid, 1-feruloyl-5-caffeoylquinic acid, 3-caffeoyl-1,5-quinolactone). On the other hand, salinity, and the combination of salinity and heat induced an upregulation of C3H, at the transcript and enzymatic activity levels, and caused an over-accumulation of these compounds (Figure 4 and Table 1).

Flavonoid synthesis starts with the condensation of p-coumaryl-CoA and three molecules of malonyl-CoA, and is catalyzed by chalcone synthase (CHS; Figure 5, Flavonoid branch pathway). Naringenin chalcone is further converted to naringenin, flavonols, and other derivatives by CHI, F3H, FLS, F3GT, and F3RT sequentially. In our experiments, transcript accumulation of *SlCHS* and *SlCHI* was upregulated under all of the stresses studied; however, the activities of their corresponding enzymes were inhibited under salinity stress, which may indicate that salinity exerted a negative post-transcriptional regulation of these enzymes. On the other hand, under the combination of salinity and heat, the activity of these enzymes and the expression of their genes showed levels that were very close to those found under control conditions, with almost no significant differences (Figure 5, Flavonoid branch pathway). Naringenin and naringenin chalcone levels under heat were significantly high with respect to control plants (Table 1). However, the concentration of these compounds under salinity or the combination of salinity and heat was lower, with this reduction being more severe under salinity alone than under salinity combined with heat (Table 1). Taken together, our findings may suggest that salinity could inhibit the accumulation of flavonols in tomato plants in favor of hydroxycinnamic acids. The expression of the *SlF3H, SlFLS, SlF3GT*, and *SlF3RT* transcripts was also downregulated under salinity, but not under heat or the combination of salinity and heat (Figure 5, Flavonoid branch pathway). This is in agreement with the concentration results of some flavonol-related compounds found in our metabolomics study, such as kaempferol, rutin, quercentin, and dehydrokaempferol, whose concentrations were higher under heat as compared to control plants, but very low under salinity (Table 1). Their concentration under salinity and heat combination was moderately higher than control plants, although still significant.

In addition to regulation at the biosynthetic level, the level of many phenolic compounds in cells is controlled by their rate of degradation, with PPO, GPX, and other peroxidases being some of the main enzymes found in plants. PPO is able to oxidize o-phenols to o-quinones, among other functions (Vaughn and Duke, 1984), and GPX is able to oxidize phenolic compounds in the presence of H_2_O_2_, thus regulating the accumulation of phenolics in plants. In our experiment, both transcript expression and enzymatic activity were strongly downregulated under all stresses applied, in agreement with the accumulation of phenolics observed in these treatments (Figure 6). However, it was not possible to establish a relationship between the preferential accumulation of hydroxycinnamic acids or flavonols and the expression and/or activity of PPO and GPX.

### Tolerance to abiotic stress could be related to flavonol synthesis and accumulation

Under abiotic stresses, ROS could be overproduced and the cell's detoxification systems could be partially or completely inhibited (Mittler, 2002; Mittler et al., 2004; Ahuja et al., 2010; Miller et al., 2010b). These detoxification systems could have an enzymatic (carried out by the redox enzymes that belong to the ascorbate-glutathione cycle) or non-enzymatic (by antioxidant compounds, such as β-carotene, ascorbic acid, anthocyanins, α-tocopherol, phenolic compounds, among others) nature. Phenolic compounds have multiple functions in plants, and among these, they have been described as powerful antioxidants (Kagan and Tyurina, 1998). The transcript expressions and enzymatic activities obtained in our oxidative metabolism study were not sufficient to explain why the oxidative damage was lower in plants grown under a combination of salinity and heat as compared to salinity alone. Our metabolomics study confirmed that most of the breakdown of lipids took place under salinity, being lower under salinity combined with heat, or heat alone. In contrast, it was also observed that the heat treatment preferentially led to the accumulation of flavonols, whereas under salinity, hydroxycinnamic acids were preferentially accumulated. The combination of both stresses resulted in the accumulation of both kinds of compounds. Taking into consideration that plants grown under heat had a lower loss of biomass than those grown under salinity or the combination of salinity and heat, we can propose that the long-term oxidative damage was lower (as our results also pointed out) in heat-treated plants as compared to the other two abiotic stress treatments. Also, taking into account that phenolic compounds can also exert antioxidant functions in plant cells, we could correlate the type of compounds that accumulated under the different types of stresses applied with the oxidative damage observed and the biomass loss obtained at the end of our experiments. Flavonols comprise the widest class of secondary metabolites, with >10,000 compounds. Among their multiple functions, flavonols are able to inhibit the generation of ROS and also quench ROS once they are formed, so they have a very important antioxidant function in cells (Agati et al., 2011; Brunetti et al., 2013). Recently, it has been shown that the biosynthesis of flavonols is upregulated under the same conditions that inactivate antioxidant enzymes, which again confirm that flavonols can act as a secondary ROS-scavenging system in plants exposed to prolonged stress conditions (Agati et al., 2011). Our results suggested that an accumulation of flavonols over other kinds of phenolic compounds may favor abiotic stress tolerance in tomato plants. To our knowledge, this is the first time that it has been shown that a differential accumulation of different types of phenolic compounds in tomato plants under different abiotic stress conditions could potentially perform a double duty: protect against oxidative damage induced by (abiotic) stress, and increase the quality of the plant product by increasing the concentration of antioxidant compounds of edible plants.

## Author contributions

TM, AG, DM, and RR performed the experimental analyses. VM and RR wrote the article with inputs from FR and RM.

## Funding

This work was supported by the Ministry of Economy and Competitiveness from Spain (GrantNo. AGL2015-66033-R), and Seneca Foundation from Region of Murcia, Spain (Grant no.15288/ PI/10).

### Conflict of interest statement

The authors declare that the research was conducted in the absence of any commercial or financial relationships that could be construed as a potential conflict of interest.

## References

[B1] Abbasi A. R. Hajirezaei M. Hofius D. Sonnewald U. Voll L. M. (2007). Specific roles of alpha- and gamma-tocopherol in abiotic stress responses of transgenic tobacco. Plant Physiol. 143, 1720–1738. 10.1104/pp.106.094771 17293434 PMC1851823

[B2] Agati G. Azzarello E. Pollastri S. Tattini M. (2012). Flavonoids as antioxidants in plants: location and functional significance. Plant Sci. 196, 67–76. 10.1016/j.plantsci.2012.07.014 23017900

[B3] Agati G. Biricolti S. Guidi L. Ferrini F. Fini A. Tattini M. (2011). The biosynthesis of flavonoids is enhanced similarly by UV radiation and root zone salinity in L. vulgare leaves. J. Plant Physiol. 168, 204–212. 10.1016/j.jplph.2010.07.016 20850892

[B4] Ahuja I. de Vos R. C. Bones A. M. Hall R. D. (2010). Plant molecular stress responses face climate change. Trends Plant Sci. 15, 664–674. 10.1016/j.tplants.2010.08.002 20846898

[B5] Almeselmani M. Deshmukh P. S. Sairam R. K. Kushwaha S. R. Singh T. P. (2006). Protective role of antioxidant enzymes under high temperature stress. Plant Sci. 171, 382–388. 10.1016/j.plantsci.2006.04.009 22980208

[B6] Anjum N. A. Sofo A. Scopa A. Roychoudhury A. Gill S. S. Iqbal M.. (2015). Lipids and proteins–major targets of oxidative modifications in abiotic stressed plants. Environ. Sci. Pollut. Res. Int. 22, 4099–4121. 10.1007/s11356-014-3917-1 25471723

[B7] Beale M. H. Ward J. L. Baker J. M. (2009). Establishing substantial equivalence: metabolomics, in Transgenic Wheat, Barley and Oats: Production and Characterization Protocols, eds Jones D. H. Shewry R. P. (Totowa, NJ: Humana Press), 289–303.

[B8] Besseau S. Hoffmann L. Geoffroy P. Lapierre C. Pollet B. Legrand M. (2007). Flavonoid accumulation in Arabidopsis repressed in lignin synthesis affects auxin transport and plant growth. Plant Cell 19, 148–162. 10.1105/tpc.106.044495 17237352 PMC1820963

[B9] Bino R. J. Hall R. D. Fiehn O. Kopka J. Saito K. Draper J.. (2004). Potential of metabolomics as a functional genomics tool. Trends Plant Sci. 9, 418–425. 10.1016/j.tplants.2004.07.004 15337491

[B10] Blokhina O. Vironlainen E. Fagerstedt K. V. (2003). Antioxidants, oxidative damage and oxygen deprivation stress: a review. Ann. Bot. 91, 179–194. 10.1093/aob/mcf118 12509339 PMC4244988

[B11] Borsani O. Zhu J. Verslues P. E. Sunkar R. Zhu J. K. (2005). Endogenous siRNAs derived from a pair of natural cis-antisense transcripts regulate salt tolerance in Arabidopsis. Cell 123, 1279–1291. 10.1016/j.cell.2005.11.035 16377568 PMC3137516

[B12] Bradford M. M. (1976). A rapid and sensitive method for the quantitation of microgram quantities of protein utilizing the principle of protein-dye binding. Anal. Biochem. 72, 248–254. 10.1016/0003-2697(76)90527-3 942051

[B13] Brennan T. Frenkel C. (1977). Involvement of hydrogen peroxide in the regulation of senescence in pear. Plant Physiol. 59, 411–416. 16659863 10.1104/pp.59.3.411 PMC542414

[B14] Brunetti C. Di Ferdinando M. Fini A. Pollastri S. Tattini M. (2013). Flavonoids as antioxidants and developmental regulators: relative significance in plants and humans. Int. J. Mol. Sci. 14, 3540–3555. 10.3390/ijms14023540 23434657 PMC3588057

[B15] Carrasco-Pozo C. Gotteland M. Speisky H. (2011). Apple peel polyphenol extract protects against indomethacin-induced damage in Caco-2 cells by preventing mitochondrial complex I inhibition. J. Agric. Food Chem. 59, 11501–11508. 10.1021/jf202621d 21954913

[B16] Chew Y. H. Halliday K. J. (2011). A stress-free walk from Arabidopsis to crops. Curr. Opin. Biotechnol. 22, 281–286. 10.1016/j.copbio.2010.11.011 21168324

[B17] Davies M. J. (2005). The oxidative environment and protein damage. Biochim. Biophys. Acta 1703, 93–109. 10.1016/j.bbapap.2004.08.007 15680218

[B18] del Rio L. A. Palma J. M. Sandalio L. M. Corpas F. J. Pastori G. M. Bueno P.. (1996). Peroxisomes as a source of superoxide and hydrogen peroxide in stressed plants. Biochem. Soc. Trans. 24, 434–438. 8736778 10.1042/bst0240434

[B19] Dorais M. Ehret D. Papadopoulos A. (2008). Tomato (*Solanum lycopersicum*) health components: from the seed to the consumer. Phytochem. Rev. 7, 231–250. 10.1007/s11101-007-9085-x

[B20] Evers D. Lefevre I. Legay S. Lamoureux D. Hausman J. F. Rosales R. O.. (2010). Identification of drought-responsive compounds in potato through a combined transcriptomic and targeted metabolite approach. J. Exp. Bot. 61, 2327–2343. 10.1093/jxb/erq060 20406784

[B21] Ferrer J. L. Austin M. B. Stewart C. Jr. Noel J. P. (2008). Structure and function of enzymes involved in the biosynthesis of phenylpropanoids. Plant Physiol. Biochem. 46, 356–370. 10.1016/j.plaphy.2007.12.009 18272377 PMC2860624

[B22] Foyer C. H. Noctor G. (2008). Redox regulation in photosynthetic organisms: signaling, acclimation, and practical implications. Antioxid. Redox Signal. 11, 861–905. 10.1089/ars.2008.2177 19239350

[B23] Foyer C. H. Shigeoka S. (2011). Understanding oxidative stress and antioxidant functions to enhance photosynthesis. Plant Physiol. 155, 93–100. 10.1104/pp.110.166181 21045124 PMC3075779

[B24] Fu J. Huang B. (2001). Involvement of antioxidants and lipid peroxidation in the adaptation of two cool-season grasses to localized drought stress. Environ. Exp. Bot. 45, 105–114. 10.1016/S0098-8472(00)00084-8 11275219

[B25] Ghasemzadeh A. Jaafar H. Z. E. Karimi E. (2012). Involvement of salicylic acid on antioxidant and anticancer properties, anthocyanin production and chalcone synthase activity in ginger (Zingiber officinale Roscoe) varieties. Int. J. Mol. Sci. 13, 14828–14844. 10.3390/ijms131114828 23203096 PMC3509612

[B26] Giovannucci E. (1999). Tomatoes, tomato-based products, lycopene, and cancer: review of the epidemiologic literature. J. Natl. Cancer Inst. 91, 317–331. 10.1093/jnci/91.4.317 10050865

[B27] Giraud E. Ho L. H. Clifton R. Carroll A. Estavillo G. Tan Y. F.. (2008). The absence of ALTERNATIVE OXIDASE1a in Arabidopsis results in acute sensitivity to combined light and drought stress. Plant Physiol. 147, 595–610. 10.1104/pp.107.115121 18424626 PMC2409015

[B28] Gomez-Romero M. Segura-Carretero A. Fernandez-Gutierrez A. (2010). Metabolite profiling and quantification of phenolic compounds in methanol extracts of tomato fruit. Phytochemistry 71, 1848–1864. 10.1016/j.phytochem.2010.08.002 20810136

[B29] Hirai M. Y. Klein M. Fujikawa Y. Yano M. Goodenowe D. B. Yamazaki Y.. (2005). Elucidation of gene-to-gene and metabolite-to-gene networks in arabidopsis by integration of metabolomics and transcriptomics. J. Biol. Chem. 280, 25590–25595. 10.1074/jbc.M502332200 15866872

[B30] Hirayama T. Shinozaki K. (2010). Research on plant abiotic stress responses in the post-genome era: past, present and future. Plant J. 61, 1041–1052. 10.1111/j.1365-313X.2010.04124.x 20409277

[B31] Iijima Y. Nakamura Y. Ogata Y. Tanaka K. I. Sakurai N. Suda K.. (2008). Metabolite annotations based on the integration of mass spectral information. Plant J. 54, 949–962. 10.1111/j.1365-313X.2008.03434.x 18266924 PMC2440531

[B32] Ju Z. Liu C. Yuan Y. (1995). Activities of chalcone synthase and UDPGal: flavonoid-3-o-glycosyltransferase in relation to anthocyanin synthesis in apple. Scientia Horticulturae 63, 175–185. 10.1016/0304-4238(95)00807-6

[B33] Kagan V. E. Tyurina Y. Y. (1998). Recycling and redox cycling of phenolic antioxidants. Ann. N.Y. Acad. Sci. 854, 425–434. 10.1111/j.1749-6632.1998.tb09921.x 9928449

[B34] Kuhl C. Tautenhahn R. Böttcher C. Larson T. R. Neumann S. (2012). CAMERA: an integrated strategy for compound spectra extraction and annotation of liquid chromatography/mass spectrometry data sets. Anal. Chem. 84, 283–289. 10.1021/ac202450g 22111785 PMC3658281

[B35] Kusano M. Redestig H. Hirai T. Oikawa A. Matsuda F. Fukushima A.. (2011). Covering chemical diversity of genetically-modified tomatoes using metabolomics for objective substantial equivalence assessment. PLoS ONE 6:e16989. 10.1371/journal.pone.0016989 21359231 PMC3040210

[B36] Kuznestov V. V. Shevyakova N. I. (1997). Stress responses of tobacco cells to high temperature and salinity. Proline accumulation and phosphorylation of polypeptides. Physiol. Plant. 100, 320–326.

[B37] Lagoa R. Graziani I. Lopez-Sanchez C. Garcia-Martinez V. Gutierrez-Merino C. (2011). Complex I and cytochrome c are molecular targets of flavonoids that inhibit hydrogen peroxide production by mitochondria. Biochim. Biophys. Acta 1807, 1562–1572. 10.1016/j.bbabio.2011.09.022 22015496

[B38] Lee D. Meyer K. Chapple C. Douglas C. J. (1997). Antisense suppression of 4-coumarate:coenzyme A ligase activity in Arabidopsis leads to altered lignin subunit composition. Plant Cell 9, 1985–1998. 10.1105/tpc.9.11.1985 9401123 PMC157052

[B39] Li P. Ma F. Cheng L. (2013). Primary and secondary metabolism in the sun-exposed peel and the shaded peel of apple fruit. Physiol. Plant. 148, 9–24. 10.1111/j.1399-3054.2012.01692.x 22989296

[B40] Macnevin W. M. Urone P. F. (1953). Separation of hydrogen peroxide from organic hydroperoxides - application to polarographic analysis of mixtures. Anal. Chem. 25, 1760–1761. 10.1021/ac60083a052

[B41] Matsuda F. Hirai M. Y. Sasaki E. Akiyama K. Yonekura-Sakakibara K. Provart N. J.. (2010). AtMetExpress development: a phytochemical atlas of arabidopsis development. Plant Physiol. 152, 566–578. 10.1104/pp.109.148031 20023150 PMC2815869

[B42] Mestre T. C. Garcia-Sanchez F. Rubio F. Martinez V. Rivero R. M. (2012). Glutathione homeostasis as an important and novel factor controlling blossom-end rot development in calcium-deficient tomato fruits. J. Plant Physiol. 169, 1719–1727. 10.1016/j.jplph.2012.07.013 22940289

[B43] Miller G. Suzuki N. Ciftci Yilmaz S. Mittler R. (2010b). Reactive oxygen species homeostasis and signalling during drought and salinity stresses. Plant Cell Environ. 33, 453–467. 10.1111/j.1365-3040.2009.02041.x 19712065

[B44] Miller G. Suzuki N. Ciftci-Yilmaz S. Mittler R. (2010a). Reactive oxygen species homeostasis and signalling during drought and salinity stresses. Plant Cell Environ. 33, 453–467. 10.1111/j.1365-3040.2009.02041.x 19712065

[B45] Mintz-Oron S. Mandel T. Rogachev I. Feldberg L. Lotan O. Yativ M.. (2008). Gene expression and metabolism in tomato fruit surface tissues. Plant Physiol. 147, 823–851. 10.1104/pp.108.116004 18441227 PMC2409049

[B46] Mittler R. (2002). Oxidative stress, antioxidants and stress tolerance. Trends Plant Sci. 7, 405–410. 10.1016/S1360-1385(02)02312-9 12234732

[B47] Mittler R. (2006). Abiotic stress, the field environment and stress combination. Trends Plant Sci. 11, 15–19. 10.1016/j.tplants.2005.11.002 16359910

[B48] Mittler R. Vanderauwera S. Gollery M. Van Breusegem F. (2004). Reactive oxygen gene network of plants. Trends Plant Sci. 9, 490–498. 10.1016/j.tplants.2004.08.009 15465684

[B49] Mittova V. Tal M. Volokita M. Guy M. (2003). Up-regulation of the leaf mitochondrial and peroxisomal antioxidative systems in response to salt-induced oxidative stress in the wild salt-tolerant tomato species *Lycopersicon pennellii*. Plant Cell Environ. 26, 845–856. 10.1046/j.1365-3040.2003.01016.x 12803612

[B50] Moco S. Bino R. J. Vorst O. Verhoeven H. A. de Groot J. van Beek T. A.. (2006). A liquid chromatography-mass spectrometry-based metabolome database for tomato. Plant Physiol. 141, 1205–1218. 10.1104/pp.106.078428 16896233 PMC1533921

[B51] Modrianský M. Gabrielová E. (2009). Uncouple my heart: the benefits of inefficiency. J. Bioenerg. Biomembr. 41, 133–136. 10.1007/s10863-009-9212-z 19365715

[B52] Mursu J. Nurmi T. Tuomainen T.-P. Salonen J. T. Pukkala E. Voutilainen S. (2008). Intake of flavonoids and risk of cancer in Finnish men: the kuopio ischaemic heart disease risk factor study. Int. J. Cancer 123, 660–663. 10.1002/ijc.23421 18338754

[B53] Nyathi Y. Baker A. (2006). Plant peroxisomes as a source of signalling molecules. Biochim. Biophys. Acta 1763, 1478–1495. 10.1016/j.bbamcr.2006.08.031 17030442

[B54] Okazaki Y. Saito K. (2012). Recent advances of metabolomics in plant biotechnology. Plant Biotechnol. Rep. 6, 1–15. 10.1007/s11816-011-0191-2 22308170 PMC3262138

[B55] Rasmussen S. Barah P. Suarez-Rodriguez M. C. Bressendorff S. Friis P. Costantino P.. (2013). Transcriptome responses to combinations of stresses in *Arabidopsis thaliana*. Plant Physiol. 161, 1783–1794. 10.1104/pp.112.210773 23447525 PMC3613455

[B56] Reznick A. Z. Packer L. (1994). Oxidative damage to proteins: spectrophotometric method for carbonyl assay. Meth. Enzymol. 233, 357–363. 8015470 10.1016/s0076-6879(94)33041-7

[B57] Rinalducci S. Murgiano L. Zolla L. (2008). Redox proteomics: basic principles and future perspectives for the detection of protein oxidation in plants. J. Exp. Bot. 59, 3781–3801. 10.1093/jxb/ern252 18977746

[B58] Rivero R. M. Kojima M. Gepstein A. Sakakibara H. Mittler R. Gepstein S.. (2007). Delayed leaf senescence induces extreme drought tolerance in a flowering plant. Proc. Natl. Acad. Sci. U.S.A. 104, 19631–19636. 10.1073/pnas.0709453104 18048328 PMC2148340

[B59] Rivero R. M. Mestre T. C. Mittler R. Rubio F. Garcia-Sanchez F. Martinez V. (2014). The combined effect of salinity and heat reveals a specific physiological, biochemical and molecular response in tomato plants. Plant Cell Environ. 37, 1059–1073. 10.1111/pce.12199 24028172

[B60] Rivero R. M. Ruiz J. M. Garcia P. C. Lopez-Lefebre L. R. Sanchez E. Romero L. (2001). Resistance to cold and heat stress: accumulation of phenolic compounds in tomato and watermelon plants. Plant Sci. 160, 315–321. 10.1016/S0168-9452(00)00395-2 11164603

[B61] Rizhsky L. Liang H. Mittler R. (2002). The combined effect of drought stress and heat shock on gene expression in tobacco. Plant Physiol. 130, 1143–1151. 10.1104/pp.006858 12427981 PMC166635

[B62] Rizhsky L. Liang H. Shuman J. Shulaev V. Davletova S. Mittler R. (2004). When defense pathways collide. The response of Arabidopsis to a combination of drought and heat stress. Plant Physiol. 134, 1683–1696. 10.1104/pp.103.033431 15047901 PMC419842

[B63] Sairam R. K. Srivastava G. C. Saxena D. C. (2000). Increased antioxidant activity under elevated temperatures: a mechanism of heat stress tolerance in wheat genotypes. Biol. Plant. 43, 245–251. 10.1023/A:1002756311146

[B64] Sandoval-Acuna C. Ferreira J. Speisky H. (2014). Polyphenols and mitochondria: an update on their increasingly emerging ROS-scavenging independent actions. Arch. Biochem. Biophys. 559, 75–90. 10.1016/j.abb.2014.05.017 24875147

[B65] Schmidt C. L. Danneel H.-J. Schultz G. Buchanan B. B. (1990). Shikimate kinase from spinach chloroplasts: purification, characterization, and regulatory function in aromatic amino acid biosynthesis. Plant Physiol. 93, 758–766. 10.1104/pp.93.2.758 16667533 PMC1062580

[B66] Serrato A. J. Perez-Ruiz J. M. Spinola M. C. Cejudo F. J. (2004). A novel NADPH thioredoxin reductase, localized in the chloroplast, which deficiency causes hypersensitivity to abiotic stress in Arabidopsis thaliana. J. Biol. Chem. 279, 43821–43827. 10.1074/jbc.M404696200 15292215

[B67] Slimestad R. Verheul M. (2009). Review of flavonoids and other phenolics from fruits of different tomato (Lycopersicon esculentumMill.) cultivars. J. Sci. Food Agric. 89, 1255–1270. 10.1002/jsfa.3605

[B68] Smith C. A. Want E. J. O'Maille G. Abagyan R. Siuzdak G. (2006). XCMS:âĂĽ processing mass spectrometry data for metabolite profiling using nonlinear peak alignment, matching, and identification. Anal. Chem. 78, 779–787. 10.1021/ac051437y 16448051

[B69] Soga T. Baran R. Suematsu M. Ueno Y. Ikeda S. Sakurakawa T.. (2006). Differential metabolomics reveals ophthalmic acid as an oxidative stress biomarker indicating hepatic glutathione consumption. J. Biol. Chem. 281, 16768–16776. 10.1074/jbc.M601876200 16608839

[B70] Sreekumar A. Poisson L. M. Rajendiran T. M. Khan A. P. Cao Q. Yu J.. (2009). Metabolomic profiles delineate potential role for sarcosine in prostate cancer progression. Nature 457, 910–914. 10.1038/nature07762 19212411 PMC2724746

[B71] Suzuki N. Rivero R. M. Shulaev V. Blumwald E. Mittler R. (2014). Abiotic and biotic stress combinations. New Phytol. 203, 32–43. 10.1111/nph.12797 24720847

[B72] Tahmasebi-Enferadi S. Rabiei Z. Vannozzi G. P. Abbas Akbari G. (2011). Shikimate dehydrogenase expression and activity in sunflower genotypes susceptible and resistant to *Sclerotinia sclerotiorum* (Lib.) de Bary. J.Agric. Sci. Tech. 13, 943–952.

[B73] Tomás-Navarro M. Vallejo F. Borrego F. Tomás-Barberán F. A. (2014). Encapsulation and micronization effectively improve orange beverage flavanone bioavailability in humans. J. Agric. Food Chem. 62, 9458–9462. 10.1021/jf502933v 25200135

[B74] Ursini F. Maiorino M. Roveri A. (1997). Phospholipid hydroperoxide glutathione peroxidase (PHGPx): more than an antioxidant enzyme? Biomed. Environ. Sci. 10, 327–332. 9315326

[B75] Vaughn K. C. Duke S. O. (1984). Function of polyphenol oxidase in higher plants. Physiol. Plant. 60, 106–112. 10.1111/j.1399-3054.1984.tb04258.x

[B76] Visioli F. Lastra C. A. D. L. Andres-Lacueva C. Aviram M. Calhau C. Cassano A.. (2011). Polyphenols and human health: a prospectus. Crit. Rev. Food Sci. Nutr. 51, 524–546. 10.1080/10408391003698677 21929330

[B77] Ward J. L. Forcat S. Beckmann M. Bennett M. Miller S. J. Baker J. M.. (2010). The metabolic transition during disease following infection of *Arabidopsis thaliana* by *Pseudomonas syringae* pv. tomato. Plant J. 63, 443–457. 10.1111/j.1365-313X.2010.04254.x 20497374

[B78] Watanabe M. Kusano M. Oikawa A. Fukushima A. Noji M. Saito K. (2008). Physiological roles of the β-substituted alanine synthase gene family in Arabidopsis. Plant Physiol. 146, 310–320. 10.1104/pp.107.106831 18024555 PMC2230570

[B79] Wu J. Howe D. L. Woodard R. W. (2003). Thermotoga maritima 3-deoxy-D-arabino-heptulosonate 7-phosphate (DAHP) synthase: the ancestral eubacterial DAHP synthase? J. Biol. Chem. 278, 27525–27531. 10.1074/jbc.M304631200 12743122

[B80] Yamaguchi T. Blumwald E. (2005). Developing salt-tolerant crop plants: challenges and opportunities. Trends Plant Sci. 10, 615–620. 10.1016/j.tplants.2005.10.002 16280254

[B81] Yamanaka T. Vincken J. P. de Waard P. Sanders M. Takada N. Gruppen H. (2008). Isolation, characterization, and surfactant properties of the major triterpenoid glycosides from unripe tomato fruits. J. Agric. Food Chem. 56, 11432–11440. 10.1021/jf802351c 18998702

[B82] Zhang Y. Butelli E. Alseekh S. Tohge T. Rallapalli G. Luo J.. (2015). Multi-level engineering facilitates the production of phenylpropanoid compounds in tomato. Nat. Commun. 6:8635. 10.1038/ncomms9635 26497596 PMC4639801

[B83] Zhu J. Fu X. Koo Y. D. Zhu J. K. Jenney F. E. Jr. Adams M. W.. (2007). An enhancer mutant of Arabidopsis salt overly sensitive 3 mediates both ion homeostasis and the oxidative stress response. Mol. Cell. Biol. 27, 5214–5224. 10.1128/MCB.01989-06 17485445 PMC1951954

