# Supplementary material for: Accumulation of Flavonols over Hydroxycinnamic Acids Favors Oxidative Damage Protection under Abiotic Stress
Source: Front Plant Sci. 2016 Jun 15;7:838. doi: 10.3389/fpls.2016.00838 (PMC4908137; doi:10.3389/fpls.2016.00838)
Supplement: Supplementary file 5 [file Table5.docx]

**Supporting Table S5.** Primers used for the quantification of the expression of the phenylpropanoid metabolism-related transcripts by qPCR.

| **GENE** | **ACCESION NUMBER (SGN)** | **FORWARD PRIMER** | **REVERSE PRIMER** |
| --- | --- | --- | --- |
| ***SlDAHPS*** | [Solyc11g009080](https://solgenomics.net/tools/blast/show_match_seq.pl?blast_db_id=224;id=Solyc11g009080.1.1;hilite_coords=173-641) | 5’caatggctctttcaagcag3’ | 5’tgggtattcgggtaactga3’ |
| ***SlSDH*** | [Solyc01g067750](https://solgenomics.net/tools/blast/show_match_seq.pl?blast_db_id=224;id=Solyc01g067750.2.1;hilite_coords=384-2028,32-327) | 5´gggaaggtggtcagtatgct3´ | 5’ttgccaagatcctcagctgt3’ |
| ***SlSK*** | [Solyc02g094420](https://solgenomics.net/tools/show_match_seq.pl?blast_db_id=224;id=Solyc02g094420.2.1;hilite_coords=683-919,417-542,539-619,332-417,247-333,172-245,116-173,624-684) | 5’aggtggaggtgcagttgttc3’ | 5’ctggcacttgcattggcata3’ |
| ***SlPAL*** | [Solyc09g007910](https://solgenomics.net/tools/show_match_seq.pl?blast_db_id=224;id=Solyc09g007910.2.1;hilite_coords=1481-1719) | 5’ggatccattgaactgggaaat3’ | 5’acctttgcagctactgaagca3’ |
| ***SlC4H*** | [Solyc01g096670](https://solgenomics.net/tools/show_match_seq.pl?blast_db_id=224;id=Solyc01g096670.2.1;hilite_coords=41-1558) | 5’ccaatggcaacgacttcaga3’ | 5’tctgcaccaaacgtccaatg3’ |
| ***Sl4CL*** | [Solyc03g117870](https://solgenomics.net/tools/blast/show_match_seq.pl?blast_db_id=224;id=Solyc03g117870.2.1;hilite_coords=966-1503) | 5’acacacaaaggcttagtcacga3’ | 5’aacagaggcaacacacacatca3´ |
| ***SlCHS*** | Solyc09g091510 | 5’atgcccgggtgtgactacc3’ | 5’ctgatgggcgaagccctag3’ |
| ***SlCHI*** | Solyc02g067870 | 5’atgccatcaattgcatgcca3’ | 5’tgtcccgaacttctccttgg3’ |
| ***SlF3H*** | Solyc06g073080 | 5’cacaccgatccaggaaccat3’ | 5’gcccaccaacttggtcttgta3’ |
| ***SlFLS*** | Solyc06g073080 | 5’gagcatgaagttgggccaat3’ | 5’tggtgggttggcctcattaa3’ |
| ***SlF3GT*** | [Solyc10g083440](https://solgenomics.net/tools/blast/show_match_seq.pl?blast_db_id=224;id=Solyc10g083440.1.1;hilite_coords=1-1335) | 5’cgaacgacgaaacactgttga3’ | 5’tgcagcatagatggcattgg3’ |
| ***SlF3RT*** | [Solyc01g094980](https://solgenomics.net/locus/12055/view) | 5’ctggcaatgcaaacagagtga3’ | 5’tcgacttgcggaagagtgaga3’ |
| ***SlPPO*** | Solyc08g074680 | 5’agaggactcattgctgagaa3’ | 5’gagtgcaacaatatcctggt3’ |
| ***SlGPX*** | Solyc08g080940 | 5’tacgctggcgataactgaact3’ | 5’atggatacaccttcaccacca3’ |
