# Supplementary material for: Accumulation of Flavonols over Hydroxycinnamic Acids Favors Oxidative Damage Protection under Abiotic Stress
Source: Front Plant Sci. 2016 Jun 15;7:838. doi: 10.3389/fpls.2016.00838 (PMC4908137; doi:10.3389/fpls.2016.00838)
Supplement: Supplementary file 6 [file Table6.docx]

**Supporting Table S6.** Primers used for quantification of the expression of the oxidative metabolism-related transcripts by qPCR.

| **GENE** | **ACCESION NUMBER (SGN)** | **FORWARD PRIMER** | **REVERSE PRIMER** |
| --- | --- | --- | --- |
| ***SlFe-SOD*** | Solyc06g048410 | 5’- taaatagagactttggttcc -3’ | 5’- tatatttgcctcttaaccct -3’ |
| ***SlCu/Zn-SOD*** | Solyc08g079830 | 5’- ggccaatctttgacccttta -3’ | 5’- agtccaggagcaagtccagt-3’ |
| ***SlcAPX*** | Solyc06g005160 | 5’- tctgaattgggatttgctga -3’ | 5’- cgtctaacgtagctgccaaa -3’ |
| ***SlCAT1*** | Solyc12g094620 | 5’- tgatcgcgagaagatacctg -3’ | 5’-cttccacgttcatggacaac-3’ |
| ***SlDHAR1*** | Solyc05g054760 | 5´- aggtggctcttggacacttc -3´ | 5´-cttcagccttggttttctgg- 3´ |
| ***SlDHAR2*** | Solyc11g011250 | 5´- cacccagagggttttgctta -3´ | 5´- ctccagtgcctgtgagatga – 3´ |
| ***SlMDHAR1*** | Solyc08g081530 | 5´- caagggtttcggttccttct-3’ | 5´- ctgcatttcctcctccaact-3’ |
| ***SlMDHAR2*** | Solyc02g086710 | 5´- agatcgttggtgcattcctc-3’ | 5´- aaaactgatgccctcctgtg-3’ |
| ***SlMDHAR3*** | Solyc09g009390 | 5´- aggaatggaatgtgctgctt-3’ | 5´- gaccgtgcctttcacaaact-3’ |
| ***SlGR1*** | Solyc09g065900 | 5’- ttggtggaacgtgtgttctt -3’ | 5’- tctcattcacttcccatcca -3’ |
