# Supplementary material for: Accumulation of Flavonols over Hydroxycinnamic Acids Favors Oxidative Damage Protection under Abiotic Stress
Source: Front Plant Sci. 2016 Jun 15;7:838. doi: 10.3389/fpls.2016.00838 (PMC4908137; doi:10.3389/fpls.2016.00838)
Supplement: Supplementary file 7 [file Table7.docx]

**Supporting Table S7.** Relative expression values of the phenylpropanoid-related transcripts. Values were normalized against Actin and EF1α as internal controls. Then, values were normalized against control samples and log_2_ was calculated and represented. Values are means of n=9

| **GENE** | **Control** | **Salinity** | **Heat** | **Salinity+heat** |
| --- | --- | --- | --- | --- |
| ***SlDAHPS*** | 0 | 3.449393 | 4.399403 | 2.100302 |
| ***SlSDH*** | 0 | 3.4949 | 3.0392 | 3.30204 |
| ***SlSK*** | 0 | 4.394993 | 3.20289493 | 2.984839 |
| ***SlPAL*** | 0 | 3.449594 | 5.204094 | 3.209399 |
| ***SlC4H*** | 0 | 2.340403 | 4.30292 | 1.967168608 |
| ***Sl4CL*** | 0 | 3.350494 | 4.20203 | 9.548309 |
| ***SlC3H*** | 0 | 4.39484933 | -3.49493 | 2.39493 |
| ***SlCHS*** | 0 | 2.24303 | 6.309392 | 3.3203 |
| ***SlCHI*** | 0 | 2.93094943 | 3.4934903 | 2.393093 |
| ***SlF3H*** | 0 | -2.378534934 | 3.20044 | 2.40320394 |
| ***SlFLS*** | 0 | -3.59303 | 4.23094 | 2.394203 |
| ***SlF3GT*** | 0 | -1.8989384 | 5.30202 | 2.3093032 |
| ***SlF3RT*** | 0 | -1.7848753 | 11.30302 | 1.192158002 |
| ***SlGPX*** | 0 | -3.35003 | -3.048383 | -3.98893 |
| ***SlPPO*** | 0 | -2.47858939 | -1.987677 | -2.989393 |
