# Supplementary material for: Accumulation of Flavonols over Hydroxycinnamic Acids Favors Oxidative Damage Protection under Abiotic Stress
Source: Front Plant Sci. 2016 Jun 15;7:838. doi: 10.3389/fpls.2016.00838 (PMC4908137; doi:10.3389/fpls.2016.00838)
Supplement: Supplementary file 8 [file Table8.docx]

**Supporting Table S8.** Relative expression values of the oxidative metabolism-related transcripts. Values were normalized against Actin and EF1α as internal controls. Then, values were normalized against control samples and log_2_ was calculated and represented. Values are means of n=9

| **GENE** | **Control** | **Salinity** | **Heat** | **Salinity+heat** |
| --- | --- | --- | --- | --- |
| ***SlFe-SOD*** | 0 | 1.73248629 | 1.10634832 | 2.39889757 |
| ***SlCu/Zn-SOD*** | 0 | 1.17248752 | 0.758729568 | 2.11869195 |
| ***SlcAPX*** | 0 | 1.23388806 | 0.915329676 | -2.05241589 |
| ***SlCAT1*** | 0 | -2.68167414 | 0.333423734 | -1.22280456 |
| ***SlDHAR1*** | 0 | -0.07856367 | 0.947105052 | 0.83995959 |
| ***SlDHAR2*** | 0 | -0.09387905 | 0.728573603 | -0.10469738 |
| ***SlMDHAR1*** | 0 | -1.12300395 | -0.027674958 | 2.22527493 |
| ***SlMDHAR2*** | 0 | -1.37016428 | -0.831877241 | 2.52381284 |
| ***SlMDHAR3*** | 0 | -0.50182127 | 0.041242982 | 1.10030491 |
| ***SlGR1*** | 0 | -1.74674272 | 0.805705185 | -2.1928254 |
