# Supplementary material for: Accumulation of Flavonols over Hydroxycinnamic Acids Favors Oxidative Damage Protection under Abiotic Stress
Source: Front Plant Sci. 2016 Jun 15;7:838. doi: 10.3389/fpls.2016.00838 (PMC4908137; doi:10.3389/fpls.2016.00838)
Supplement: Supplementary file 9 [file Table9.docx]

**Supporting Table S9.** Absolute values obtained for the enzymatic activities of the phenylpropanoid metabolism-related enzymes. Values are expressed as µmol of product (mg prot)^-1^(min)^-1^ ± SE (n=6).

| **ENZYME** | **Control** | **Salinity** | **Heat** | **Salinity+heat** |
| --- | --- | --- | --- | --- |
| **DAHPS** | 9.24 ± 0.323 | 25.47 ± 0.891 | 59.56 ± 2.084 | 440.42 ± 15.41 |
| **SK** | 3.21 ± 0.112 | 13.13 ± 0.459 | 39.61 ± 1.38 | 171.26 ± 5.99 |
| **SDH** | 0.312 ± 0.010 | 1.38 ± 0.048 | 13.13 ± 0.459 | 70.84 ± 2.47 |
| **PAL** | 27.21 ± 0.952 | 156.23 ± 5.46 | 617.02 ± 21.59 | 4181.24 ± 146.3 |
| **C4H** | 21.14 ± 0.740 | 239.98 ± 8.39 | 2963.4 ± 103.72 | 36435.7 ± 1275 |
| **4CL** | 8.23 ± 0.288 | 34.76 ± 1.21 | 147.34 ± 5.15 | 639.33 ± 22.37 |
| **C3H** | 0.35 ± 0.012 | 1.14 ± 0.039 | 3.87 ± 0.13 | 9.80 ± 0.34 |
| **CHS** | 254.15 ± 8.89 | 2370.1 ± 82.9 | 360.53 ± 12.61 | 1165.5 ± 40.79 |
| **CHI** | 2.54 ± 0.08 | 0.594 ± 0.020 | 3.85 ± 0.134 | 5.19 ± 0.181 |
| **F3H** | 54.69 ± 1.91 | 10.06 ± 0.352 | 111.08 ± 3.88 | 174.50 ± 6.10 |
| **F3GT** | 0.526 ± 0.018 | 0.069 ± 0.002 | 0.584 ± 0.020 | 0.797 ± 0.027 |
| **GPX** | 9.25 ± 0.323 | 3.22 ± 0.11 | 33.51 ± 1.17 | 53.49 ± 1.87 |
| **PPO** | 11.21 ± 0.392 | 11.64 ± 0.407 | 0.128 ± 0.004 | 0.023 ± 0.0008 |
