# Supplementary material for: Accumulation of Flavonols over Hydroxycinnamic Acids Favors Oxidative Damage Protection under Abiotic Stress
Source: Front Plant Sci. 2016 Jun 15;7:838. doi: 10.3389/fpls.2016.00838 (PMC4908137; doi:10.3389/fpls.2016.00838)
Supplement: Supplementary file 10 [file Table10.docx]

**Supporting Table S10.** Absolute values obtained for the enzymatic activities of the oxidative metabolism-related enzymes. Values are expressed as µmol of product (mg prot)^-1^(min)^-1^ ± SE (n=6).

| **GENE** | **Control** | **Salinity** | **Heat** | **Salinity+heat** |
| --- | --- | --- | --- | --- |
| **SOD** | 5.42956168 | 17.1220314 | 12.9495524 | 16.6783046 |
| **CAT** | 10.8265435 | 3.59458914 | 13.8430801 | 5.7645774 |
| **APX** | 14.5444477 | 28.9981432 | 21.4046062 | 6.2060522 |
| **DHAR** | 24.6315049 | 10.6548203 | 58.89631 | 5.3734646 |
| **MDHAR** | 136.631088 | 16.9228366 | 248.511209 | 385.929772 |
| **GR** | 6.30815563 | 1.43040744 | 6.90986154 | 3.12434661 |
