# Supplementary material for: Accumulation of Flavonols over Hydroxycinnamic Acids Favors Oxidative Damage Protection under Abiotic Stress
Source: Front Plant Sci. 2016 Jun 15;7:838. doi: 10.3389/fpls.2016.00838 (PMC4908137; doi:10.3389/fpls.2016.00838)
Supplement: Supplementary file 11 [file Image1.pdf]

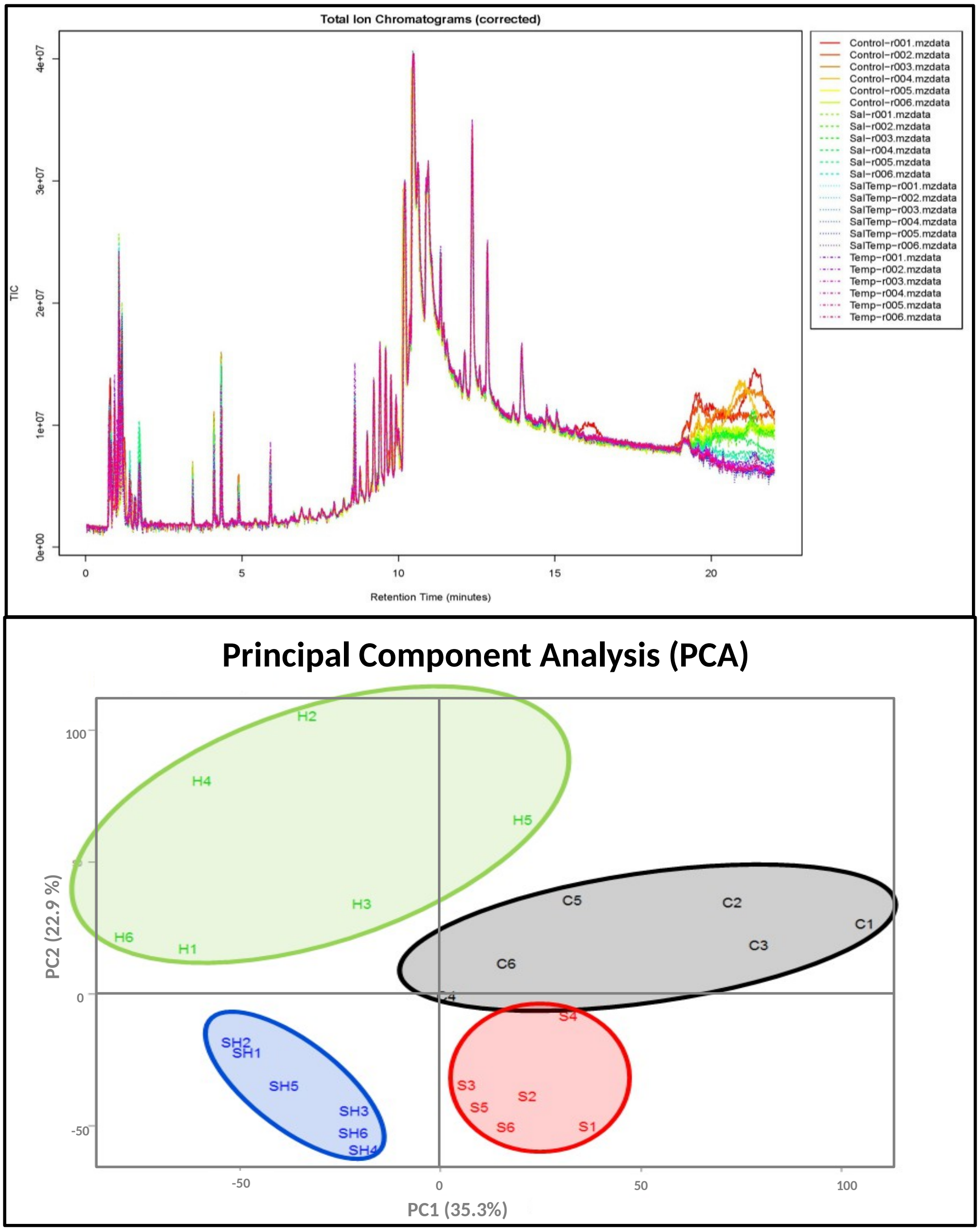

Supplemental Figure 1. Total ion chromatogram (A) and principal component analysis of the metabolomics study. Toamto leaves grown under control (C1-C6), salinity (S1-S6) Heat (H1-H6) and the combination of salinity + heat (SH1-SH6) were subjected to a metabolomics study in a UPLC-QTOF (Agilent 6550) and total ion chromatogram of the different samples is shown (A). A PCA analysis showing the variation between biological samples and treatments was conducted in this study.
