# Supplementary material for: Accumulation of Flavonols over Hydroxycinnamic Acids Favors Oxidative Damage Protection under Abiotic Stress
Source: Front Plant Sci. 2016 Jun 15;7:838. doi: 10.3389/fpls.2016.00838 (PMC4908137; doi:10.3389/fpls.2016.00838)
Supplement: Supplementary file 12 [file Image2.pdf]

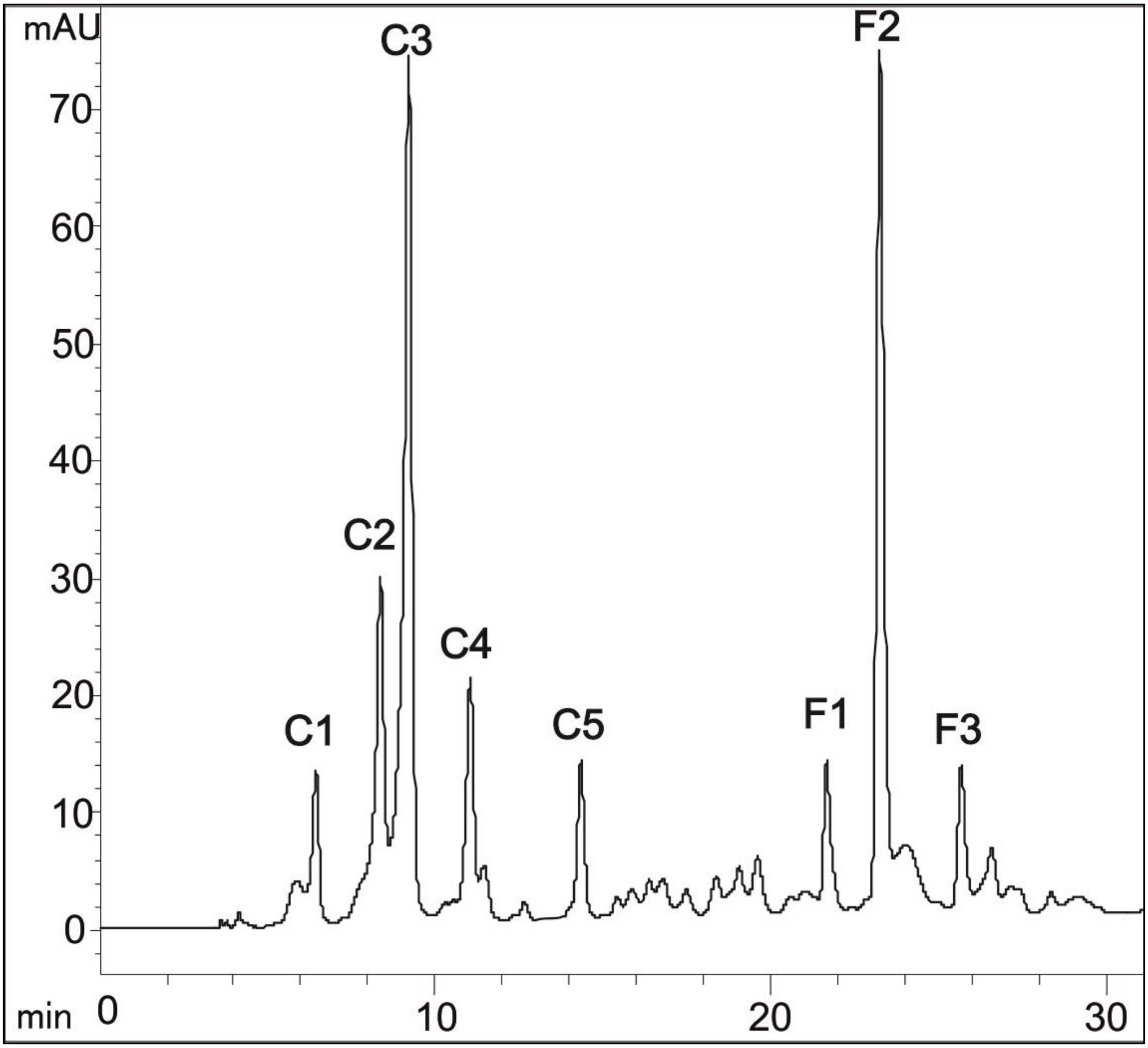


**Supplemental Figure 2.** Tomato leaves cromatograms for phenols detection by HPLC. C1-C5: chlorogenic acids detected. F1-F3: Flavonols detected. Samples were extracted and injected as indicated in Material and Methods section.
